# Poorly quantified trends in ammonium nitrate remain critical to understand future urban aerosol control strategies

**DOI:** 10.1126/sciadv.adt8957

**Published:** 2025-05-21

**Authors:** Ryan X. Ward, Haroula D. Baliaka, Benjamin C. Schulze, Gaige H. Kerr, John D. Crounse, Sina Hasheminassab, Roya Bahreini, Ann M. Dillner, Armistead Russell, Nga L. Ng, Paul O. Wennberg, Richard C. Flagan, John H. Seinfeld

**Affiliations:** ^1^ California Institute of Technology, Pasadena, CA, USA.; ^2^ George Washington University, Washington, DC, USA.; ^3^ Jet Propulsion Laboratory, Pasadena, CA, USA.; ^4^ University of California, Riverside, Riverside, CA, USA.; ^5^ University of California, Davis, Davis, CA, USA.; ^6^ Georgia Institute of Technology, Atlanta, GA, USA.; ^7^ Environmental Defense Fund, New York, NY, USA.

## Abstract

Despite decades of progress in reducing nitrogen oxide (NO*_x_*) emissions, ammonium nitrate (AN) remains the primary inorganic component of particulate matter (PM) in Los Angeles (LA). Using aerosol mass spectrometry over multiple years in LA illustrates the controlling dynamics of AN and their evolution over the past decades. These data suggest that much of the nitric acid (HNO_3_) production required to produce AN in LA occurs during the nighttime via heterogeneous hydrolysis of N_2_O_5_. Further, we show that US Environmental Protection Agency–codified techniques for measuring total PM_2.5_ fail to quantify the AN component, while low-cost optical sensors demonstrate good agreement. While previous studies suggest that declining NO*_x_* has reduced AN, we show that HNO_3_ formation is still substantial and leads to the formation of many tens of micrograms per cubic meter of AN aerosol. Continued focus on reductions in NO*_x_* will help meet the PM_2.5_ standards in the LA basin and many other regions.

## Teaser

Regulatory monitors fail to measure ammonium nitrate aerosol, masking its importance as a top aerosol pollutant in Los Angeles.

## INTRODUCTION

The Los Angeles (LA) basin is one of the most polluted urban airsheds in the United States. LA County has failed to attain the US Environmental Protection Agency’s (EPA) 2012 National Ambient Air Quality Standard (NAAQS) for PM_2.5_ (Particulate Matter less than 2.5 μm in diameter) every year in the past decade (*1*). In 2024, the standard was strengthened from 12 to 9 μg m^−3^ annually averaged (*2*), posing even more urgent regulatory pressure. This pollution is not necessarily unusual, as a confluence of physical and anthropogenic phenomena prime the LA basin for poor air quality: a large population, favorable meteorology, geographic barriers for ventilation, and an abundance of natural and anthropogenic sources of emissions, to name a few (*3*). It is unexpected that, given the reductions in precursor emissions (like NO*_x_*), the PM levels have stagnated over the past decade.

The PM in LA is largely produced by the partitioning of gaseous species produced in the atmosphere to the condensed phase (so-called secondary PM). Further, the PM contains a variety of organic and inorganic constituents. Given the complicated processes that tie the emissions of volatile organic and inorganic compounds to the formation of secondary PM, determining the relative importance of these emission sources remains a challenge and hinders opportunities for abatement (*4*, *5*). Although recent studies have focused on characterizing and apportioning the organic component of secondary aerosol (*6*–*10*), inorganic species (primarily secondary nitrate and sulfate) contribute most of the PM_2.5_ mass in LA and much of Southern California (*11*).

Ammonium nitrate (NH_4_NO_3_; AN) has long been identified as one of the key components of LA aerosol (*11*, *12*). The particulate nitrate is produced from the copartitioning of nitric acid (HNO_3_) with ammonia (NH_3_). HNO_3_ forms in the atmosphere from nitrogen oxides (NO*_x_*), whose source is overwhelmingly combustion of fossil fuels in automobiles, trucks, and off-road engines. NH_3_ is emitted from agricultural, vehicular, and industrial processes and is the predominant base in most urban and rural settings (*13*). The resulting AN maintains a delicate balance between the particle and the gas-phase HNO_3_ and NH_3_. Unlike the other inorganic constituents of PM (e.g., ammonium sulfate), AN is readily evaporated from the aerosol (as NH_3_/HNO_3_), and its partitioning is constrained by many factors, such as relative humidity (RH) and temperature (*14*). Despite many decades of progress in emission abatement, specifically in reducing NO*_x_*, many urban airsheds are still plagued by AN (*11*, *15*–*17*), posing risk to human health (*18*).

Given its prominence, it is critical from a regulatory perspective to understand the trends and formation mechanisms of AN. In part, there is an expectation that as particulate sulfate concentrations decrease, ammonium is freed to neutralize nitrate, thus increasing its formation potential in urban settings (*19*, *20*, *21*). In LA, sulfate concentrations have declined over the past two decades (*22*), so it is expected that the importance of the AN is likely increasing relative to ammonium sulfate or bisulfate. Of course, the trend in AN formation will be limited by trends in other factors, such as available oxidants (OH, O_3_, etc.), aerosol surface area, aerosol liquid water, and its precursors NO*_x_* and NH_3_. Since AN is not directly emitted, identifying the precursor sensitivity is key for generating a control strategy, and studies have suggested that, depending on the chemical and meteorological regime, particulate nitrate can be preferentially sensitive to either NO*_x_* or NH_3_ reductions (*23*–*27*). While NH_3_ emissions have been linked to limitations in AN formation in LA (*28*), this sensitivity is likely dynamic, and it is itself a superposition of the trends in all the factors contributing to AN formation. NH_3_ emissions characterized by bottom-up inventories have remained relatively constant or increased over the past two decades (see fig. S1), and top-down evidence has suggested similar trends in NH_3_ globally (*29*, *30*). Since NO*_x_* emissions and atmospheric burdens have simultaneously declined, naive intuition says that the limiting reagent for AN formation (i.e., NH_3_ or NO*_x_*) is trending toward NO*_x_* on the basis of reactant abundance.

Unfortunately for regulators, AN is one of the most difficult PM species to measure because of its temperature-sensitive thermodynamic equilibrium. It is well documented that AN can evaporate during PM sampling (*31*, *32*). Relatively small changes in temperature or partial pressure can rapidly evaporate the AN from a filter, which is the EPA’s Federal Reference Method (FRM) standard for sampling PM (*31*, *33*–*35*). During the 1990s in LA, this artifact was measured to potentially exceed 10 μg m^−3^ on a single day (*32*), essentially all of the nitrate mass, thereby introducing a substantial negative bias in the total PM_2.5_ (a regulated quantity). More recently, Chiu and Carlton (*35*) pointed out that this volatilization exists across the state of California, appreciably influencing the FRM measurements of PM_2.5_ over the past two decades. Observations reported here illustrate that AN concentrations in PM_2.5_ often vastly exceed those previously reported by mass spectral methods in LA, suggesting that our previous understanding of the magnitude of nitrate formation in LA is underestimated.

In this study, we (i) document the persistence of AN as a ubiquitous constituent of LA aerosol and (ii) explore the pathologies to particulate nitrate sampling that have obscured the important contribution of AN to the total PM mass. Using an advancement in the design of instrumentation to enable high–time resolution chemical analysis of the entire PM_2.5_, we revisit our understanding of nitrate production and measurement in the LA basin.

## RESULTS

### Persistence of AN PM in LA

**Fig. 1.**
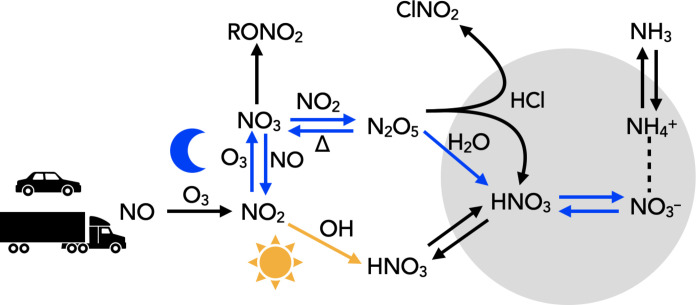
Schematic of the fate of NO_2_ in an urban environment leading to particle phase ${\text{NO}}_{3}^{-}$ formation. The yellow pathway is the primary daytime formation route for HNO_3_; the blue pathway represents the primary nighttime formation mechanism of HNO_3_ we consider in this work. The gray represents aerosol.

HNO_3_ is the essential ingredient leading to the formation of AN aerosol. It is produced via two major pathways: a daytime, OH-initiated mechanism and a nighttime, O_3_-initiated mechanism. In Fig. 1, we highlight both of these pathways schematically. During the day, the OH-initiated oxidation of NO_2_ forms HNO_3_ directly in the gas phase. At night, the formation of N_2_O_5_ can proceed uninhibited by the photolysis of the NO_3_ radical. In the presence of aerosol water (and sufficient surface area), N_2_O_5_ is hydrolyzed to form HNO_3_ in the particle phase (as opposed to the gas phase in the daytime mechanism). The partitioning of HNO_3_ with the aerosol phase is heavily influenced by NH_3_, which raises aerosol pH and generates a thermodynamic equilibrium favoring aerosol-phase nitrate.

**Fig. 2.**
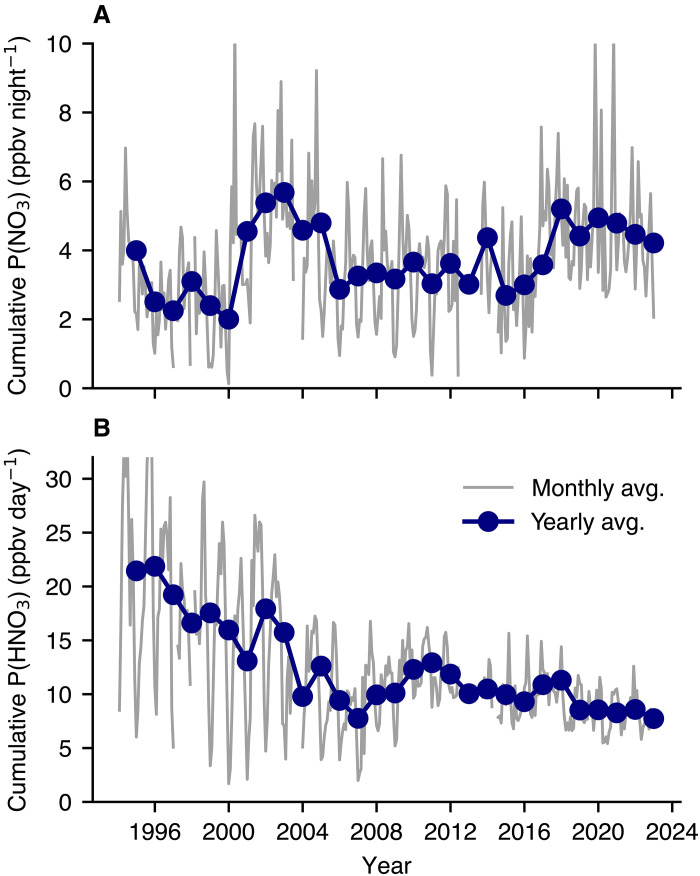
Decadal NO_3_ trends in Pasadena. (**A**) Estimated trend in overnight nitrate radical production in Pasadena. Nighttime production results only from oxidation of NO_2_ by O_3_; this rate is summed over each night to calculate cumulative NO_3_ production. This represents the maximum amount of HNO_3_ produced via N_2_O_5_ hydrolysis. Loss of NO_3_ via reaction with alkenes to form organic nitrates or reaction of NO with NO_3_ will reduce the amount of HNO_3_ formed at night. (**B**) Estimated trend in a proxy for the cumulative daytime nitric acid production in Pasadena. Daytime HNO_3_ results from the oxidation of NO_2_ by OH, the former of which is measured and the latter of which is parameterized by its relationship with NO. Note the difference in *y*-axis scale (roughly a factor of 3). ppbv, parts per billion by volume.

We calculate that, over the past three decades, the formation of HNO_3_ (and thus AN) is increasingly influenced by the nighttime pathway. In urban settings with very high emissions of NO, the reaction of NO with O_3_ can titrate O_3_ at night, inhibiting the formation of the NO_3_ radical (*14*, *36*). Reflecting these dynamics, decades of reductions in NO emissions in LA have increased nighttime O_3_ by nearly a factor of 3 since 2000 (see fig. S2). Nighttime NO_3_ radical production depends on both NO*_x_* and O_3_, so the decline in NO*_x_* is largely compensated by the increase in nighttime O_3_. During the day, decreasing NO*_x_* concentrations should similarly cause a decrease in daytime HNO_3_ production, but even here, there is compensation as OH levels have increased as NO_2_ decreased (*37*, *38*). We estimate the trends in the relative formation pathways for the particulate nitrate precursors (namely gas-phase HNO_3_ during the day and NO_3_ radical at night), shown in Fig. 2. The nighttime production of NO_3_ radical, calculated in Fig. 2A as the cumulative overnight production (the total amount of NO_3_ produced in a single night), has in general remained nearly constant, meaning that declining NO*_x_* concentrations compensate for increasing nighttime O_3_ concentrations. We estimate that the daytime formation of HNO_3_, which we calculate via a proxy for OH exposure (see section S3.2), has fallen by more than half over the past 25 years; the sign of this trend is consistent with observational evidence (*39*). These calculations, in concert with declining sulfate concentrations over the same time span (which frees ${\text{NH}}_{4}^{+}$ for interaction with ${\text{NO}}_{3}^{-}$), suggest that the importance of the nighttime mechanism has increased relative to the daytime mechanism (*21*, *22*).

Keeping the precursor trend in mind, we turn to measurements of aerosol nitrate. We present here quantitative mass spectral measurements of the real-time, nonrefractory PM_2.5_ composition in LA. These observations are made possible with the development of the Aerodyne aerosol chemical speciation monitor (ACSM) with a PM_2.5_ aerodynamic lens inlet, enabling long-term quantification of the aerosol composition up to 2.5 μm in diameter, unlike the aerosol mass spectrometer (AMS) that typically has a PM_1_ lens (*40*–*42*). We obtained observations using the ACSM in the springtime of 2023 in Pasadena and during the summer and fall of 2023 in Pico Rivera, both of which are urban locations downwind of the LA urban core.

**Fig. 3.**
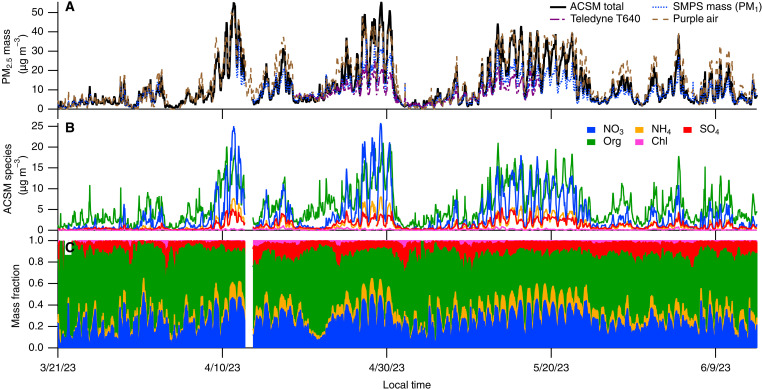
Pasadena Spring 2023 PM measurements. Time series of data from various instruments in Pasadena during spring and early summer of 2023. (**A**) shows total particle mass measurements, while (**B**) and (**C**) are speciated mass concentrations and mass fractions from the ACSM.

Figure 3 and fig. S3 show time series measurements of the ACSM data from both locations (in addition to data from other PM monitors); immediately notable is the timing of the aerosol-phase nitrate throughout the year, which generally peaks at night/early morning and often at magnitudes exceeding 10 μg m^−3^. Compare these measurements to observations of PM_2.5_ made in the 1970s (*43*), which display morning and afternoon peaks in particulate nitrate at concentrations of similar magnitude to those observed in Pico Rivera (e.g., 30 μg m^−3^ for a 2-hour average). The 1970s observations feature a prominent afternoon peak that matches the timing of transport and photochemical processing from downwind of Downtown LA. In contrast, in 2009 and 2010, measurements from an AMS (PM_1_) do not show this afternoon peak in particulate nitrate; instead, only an uptick in nitrate aerosol after sunset and an early morning peak are observed, consistent with a nighttime chemical production mechanism and favorable thermodynamics (i.e., lower temperatures and higher RH at night) (*5*, *44*). A similar trend is observed in 2022 AMS data, although nitrate concentrations are smaller (see fig. S4). In summary, since the 1970s, the formation of particulate nitrate has shifted from a predominantly daytime process to a nighttime mechanism, and the amount of AN formed has declined with declining NO*_x_* emissions in the basin.

It is instructive to think about the size partitioning of the AN—that is, which size fraction of the PM bears the brunt of the AN mass. All previous mass spectral measurements of aerosol in LA were made using the Aerodyne AMS (a PM_1_ measurement), that is in both the 2009 and 2010 campaigns (PACO and CalNex) and in the 2021 and 2022 campaigns (RECAP, LAAQC, and CalNexT). Submicrometer aerosol loadings of more than 10 μg m^−3^ of particulate nitrate (hourly average) were never observed by the AMS (*5*, *45*). In contrast, despite very similar meteorology in 2023, the ACSM often saw nitrate in excess of 10 μg m^−3^. We suggest that the major difference is that, when nitrate is highly elevated, a substantial fraction of the AN is found on particles larger than 1.0 μm that are, therefore, not observed by the PM_1_-AMS. It would not necessarily be unexpected if much of the past LA PM_2.5_ mass was found on particles smaller than 1 μm, meaning that the AMS (or other PM_1_ measurement techniques) provide a reasonable estimate of the PM_2.5_ mass. This has been observed with the PM_2.5_ ACSM at other locations, such as the wintertime San Joaquin Valley and urban Atlanta (*46*, *47*). However, mass closure with another particle sampling instrument, the scanning mobility particle sizer (SMPS, a PM_1_ measurement), and the ACSM (as noted before, a PM_2.5_ measurement) suggests that for spring of 2023, there is a sizable amount of mass in PM_1–2.5_ and that it is made largely of AN (see figs. S5 and S6). Our hypothesis that there is an enhancement of secondary nitrate in larger particles is supported both theoretically (Kelvin constraints) and observationally (*5*, *48*–*50*). We note that the SMPS could possibly introduce drying artifacts that would bias its measurement low (*51*); furthermore, while the SMPS upper size bound is 740 nm, it has been shown to broadly capture LA PM_1_ mass (*5*). In addition, the ACSM observations are consistent with corrected filter observations of PM_2.5_, adding credence to their validity (see the next paragraph and fig. S7).

To investigate further the large particulate nitrate observations, we estimated the production of particle-phase nitrate from available precursors (namely NO_2_ and O_3_ at night). In brief (see section S3.1 for a more rigorous development), we calculate the total production of particulate nitrate from its precursors over a single night and compare the results with the ACSM observed growth in particulate nitrate. Essentially, we are testing this question: If transport were not important (that is, all particulate nitrate were formed locally at night), and if the conversion of NO_3_ radical to particle phase nitrate were 100% efficient (that is, it follows the blue arrows in Fig. 1), can the gas-phase observations explain the particle-phase observations? We focus primarily on the second assumption since we believe that the first approximation is reasonable given the nighttime wind speeds in LA during the measurement periods [see section S6 for conditional probability function (CPF) analysis].

**Fig. 4.**
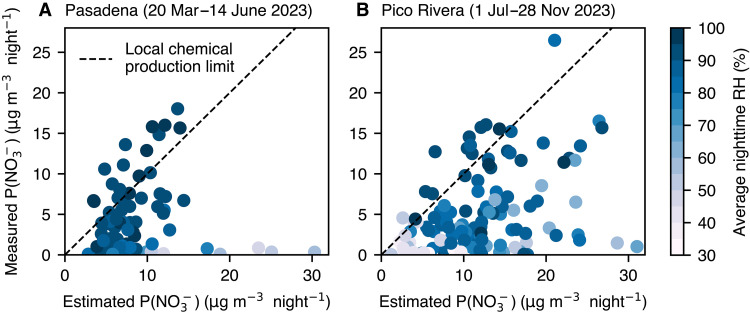
Estimated and measured production of ${\text{NO}}_{3}^{-}$ aerosol for each night in springtime Pasadena and summer/fall Pico Rivera. Each data point represents one night in (**A**) springtime Pasadena and (**B**) summer and fall in Pico Rivera. The measurements were made by the ACSM (maximum overnight value observed minus the sunset value), and the overnight production estimates were calculated from precursor concentrations (namely, NO_2_ and O_3_). The dashed line represents the “local chemical production limit” (that is, all NO_3_ is locally converted to ${\text{NO}}_{3}^{-}$). Points are colored by the average measured nighttime RH.

The comparison of predicted and observed nitrate formation is shown in Fig. 4 for the Pasadena and Pico Rivera datasets. On many nights, the particulate nitrate is, to good approximation, explained by the estimated NO_3_ radical production. As shown in Fig. 4, the comparison between the production of NO_3_ and the increase in the amount of AN scatter around the 1:1 line—behavior we call the “local chemical production limit.” In Pasadena, even when sufficient NO_2_ and O_3_ are present to create abundant N_2_O_5_, there are often nights when elevated AN is not observed. These times are generally when the RH is low (the gray points in Fig. 4A), consistent with inefficient hydrolysis of N_2_O_5_. The data from Pico Rivera tend to show more diversity in results, although when high nitrate is observed, it is almost always explained by the nighttime formation mechanism. The Pico Rivera dataset covers a much longer time period than the observations in Pasadena, and therefore a broader suite of meteorological and chemical conditions (e.g., production of organic nitrates or nitryl chloride, or limitations by NH_3_) that may depress the formation of nitrate aerosol (*28*). We extended this analysis to other field campaigns, including CalNexT and LAAQC (see section S1.4); in general, we observe that, in spring and early summer, slow hydrolysis is the primary constraint on AN formation (see fig. S8), while in late summer, temperature (and, therefore, partitioning of NH_3_ and HNO_3_ to the gas phase) plays an important role in limiting its formation (see fig. S9).

Regardless of season, the nighttime formation pathway has the potential to form more than 30 μg m^−3^ of secondary nitrate given the amount of NO*_x_* in the LA basin, comparable to observations in urban Beijing (*16*, *52*). Our analysis shows that there are ample precursors (namely NO_2_, O_3_, and NH_3_) present to account for the ACSM observations and that these concentrations are much higher than what has been reported over the past decade by other mass spectral measurements.

From a regulatory perspective, NO*_x_*, and therefore mobile sources, remain extremely critical contributors to secondary aerosol production. Their contributions are often on par with or exceed that of the organic component of the aerosol, which has typically been considered to dominate secondary aerosol in LA (*7*, *10*). While controlling secondary aerosol formation is not trivial, the link between nitrate aerosol and its precursors is much better constrained than that of organic aerosol, suggested in part by this work. In springtime Pasadena especially, reductions in NO*_x_* can still serve as an effective control strategy for PM_2.5_ and are on par or more important than reductions in NH_3_. Depending on the meteorological and chemical regime, though, both can likely serve as controls on AN in LA, and we discuss this sensitivity further in the Supplementary Materials (section S4).

### PM measurement pathologies driven by AN

**Fig. 5.**
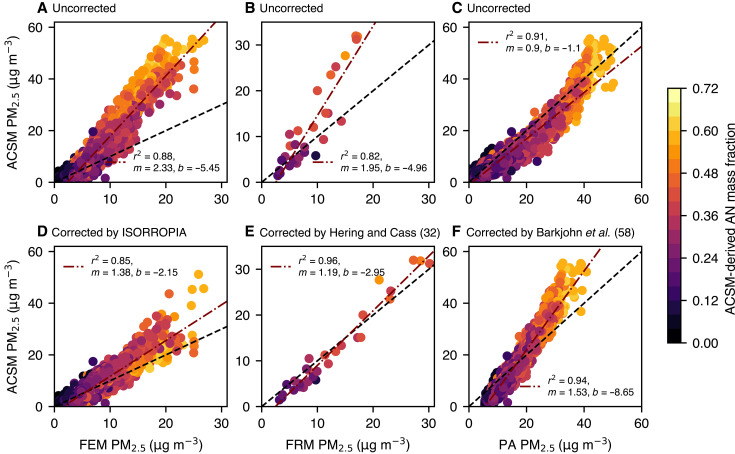
Comparison of PM_2.5_ measurements in Pasadena during the spring of 2023. (**A**) to (**C**) scatter uncorrected measurements, while (**D**) to (**F**) show corrections made to the data as described in the Supplementary Materials. The FEM and PurpleAir (PA) comparisons are hourly averages, while the FRM is a daily average (measured every third day). Data points are colored by the ACSM-derived mass fraction of AN. The dash-dot lines are fits to the data, and the dashed lines are 1:1 lines.

Given that AN remains such a critical pollutant in LA, the limited contemporary discussion of its role is unexpected. In large measure, this reflects the challenges in its quantification. A comparison of PM_2.5_ measurement instruments for the 2023 Pasadena dataset is shown in Fig. 5. The ACSM measures substantially more PM_2.5_ than either the FRM or Federal Equivalence Method [(FEM) here, a Teledyne T640] techniques; further, the difference can be attributed almost entirely to AN (i.e., larger discrepancies tend to track more AN in the aerosol). Figure 5 (A and B) illustrates that the codified EPA techniques for PM_2.5_ compliance monitoring do not accurately reflect the aerosol burden in LA—substantially underreporting the PM_2.5_ mass. In contrast, the low-cost optical sensor (PurpleAir) most consistently tracks the ACSM observations, as shown in Fig. 5C. This likely reflects preservation of AN before measurement by the low-cost sensor. In contrast, the FRM (Teflon filter) and the FEM (which heats the sample before optically measuring the particle scattering) are less likely to observe AN due to evaporation.

While it is not possible to know the amount of AN measured by the FEM, FRM, or PurpleAir, we can reverse engineer what would be expected from these sensors using the ACSM data after accounting for AN volatilization. In the case of the FRM, an empirical parameterization was designed in 1999 for these measurements and verified for PM in California (*32*, *35*), and we apply this parameterization, shown in Fig. 5E. There is a dramatic improvement in the correlation and slope between the ACSM and FRM [coefficient of determination (*r*^2^) = 0.96 and *m* = 1.19 over the measurement period], suggesting that almost all of the bias is driven by the volatilization of AN. The Hering *et al.* (*32*) temperature-based parameterization (constrained by the true observations of nitrate; here the ACSM) appears to capture AN loss accurately even when much of the production happens at night, when temperatures are cooler. The parameterization suggests that sufficient temperature-driven volatilization occurs over the 24-hour sample period to drive a large reduction in nitrate (sometimes all of the nitrate produced).

In the case of the FEM, we use the ISORROPIA model to correct the data for loss of AN (see section S2.2 for details). This Teledyne FEM instrument heats the sample line to achieve a 35% RH for sampling, which shifts the AN equilibrium into the gas phase. After accounting for the evaporation of AN, the *r*^2^ does not increase (0.88 uncorrected, 0.85 corrected), but the slope of the regression between the two instruments is much closer to 1 (2.33 uncorrected, 1.38 corrected)—again consistent with the hypothesis that volatilization of AN is responsible for most of the difference between the ACSM and FEM measurements. Evaporation of other semivolatile species, such as secondary organics, could play a role in the remaining difference.

The EPA and Teledyne released a joint statement in 2024 suggesting that the FEM measurements must be adjusted to achieve better agreement between the FEM and FRM data (*53*). We detail these calculations and apply them to our data in the Supplementary Materials (section S2.3) (*54*). For all PM_2.5_ concentrations and temperatures, this correction leads to a further reduction in the PM_2.5_ reported from the Teledyne instrument. Previous work suggests that, across the EPA sites using these instruments, both positive and negative biases relative to the FRM are found (*55*). In fig. S13 (E and F), we illustrate the impact of the suggested correction. The comparison between the FEM and the FRM does not improve. Further, because this correction reduces the Teledyne measured value, the artifact introduced by AN volatilization is made worse. Our data illustrate the importance of having robust knowledge of the aerosol composition and size for understanding discrepancies between measurements of PM from different methods and instruments. This effort to improve the agreement between the FEM and the FRM (and other techniques) suggests a tension between accurately reflecting the state of the atmosphere and adjusting a measurement output to match a regulatory standard (the PM_2.5_ FRM). Resolving this tension will be difficult given that the legal framework for regulatory compliance rests on an analytical method that is fundamentally flawed in its ability to measure a key component of atmospheric aerosol.

In a similar vein to the FEM, many models (e.g., EPA’s air quality model, CMAQ) and datasets use FRM data to benchmark model performance (*56*). We compare a subset of the CMAQ EQUATES data (see section S8) with FRM data collected in Pasadena, shown in fig. S14 (*57*). Having established that the FRM does not accurately capture AN, the agreement between the simulations and observations seems to be spurious, suggesting that the regulatory models inadequately capture the production of secondary aerosol in the LA basin or in other locations where AN is common. We discuss this further in the Supplementary Materials.

Perhaps one silver lining of this analysis is the performance of the PurpleAir sensor (Fig. 5C); while it shows nonlinearity, its uncorrected data best captures the range of PM magnitudes observed by the ACSM. Furthermore, corrections applied to the dataset, which are themselves derived from PurpleAir/FRM comparisons (*58*), do not improve the performance of the sensor (the regressed slope goes from 0.9 to 1.53, a departure from 1), although there are a diversity of calibration types and more complex data analysis schemes that may improve its skill (*59*, *60*). There is a physical basis for correcting optical sensors during periods of elevated RH (due to increased light scattering by liquid water) (*58*), but these data suggest that, at least for Pasadena in spring of 2023, this correction is minor. From a regulatory perspective, it is important to recognize that, unlike the FRM/FEM products, the PurpleAir sensor is likely less susceptible to evaporation artifacts, particularly those due to AN, and so they are perhaps more suitable for deployment in regions of high AN pollution like Southern California. Further, they are online measurements and require little supervision compared to the FRM, which is a labor-intensive technique. Higher quality optical particle counters that accurately capture the size distribution in the 1- to 2.5-μm size range would likely show even better agreement with the ACSM.

In general, the interferences to instrumental measurements introduced by AN partitioning and their impacts on ambient data interpretation are well documented. Care is required when interpreting data for these species (*61*, *62*). For instance, it has previously been observed that particulate nitrate can affect measurements of gas-phase HNO_3_ detection (*63*). We highlight this artifact in contemporary measurements of HNO_3_ by collocated chemical ionization mass spectrometers, detailed in the Supplementary Materials (section S1.3) and fig. S15.

While we do not necessarily advocate for the substitution of an ACSM or PurpleAir sensor in lieu of the FRM/FEM monitors, we do highlight the importance of speciated PM measurements. In LA, filter-based, speciated PM measurements exist at some locations; these measurements use nylon filters instead of Teflon to minimize evaporative losses, as used at CSN and IMPROVE sites. The ACSM measurements over the spring Pasadena measurement period, while not exactly collocated with nylon filters, compare well to sites in the area (see fig. S7). To be clear, it is unexpected that decades after Hering *et al.* (*32*) demonstrated that nylon filters are much more effective than the FRM or FEM for quantifying PM, the FRM has not adopted this approach. As newer, online techniques become widely available for real-time monitoring, it may be time to reconsider our approach for long-term measurements for regulatory compliance.

## DISCUSSION

Poor air quality in the LA basin reflects the persistent role of NO*_x_* despite decades of reductions in its emissions. In the equinox seasons, NO_2_ very effectively forms nitrate aerosol. This suggests that continued effort to reduce NO*_x_* emissions is essential for meeting regulatory goals. Unfortunately, more than 80% of NO*_x_* emissions in the basin (largely from mobile sources) are not regulated at the local level, making efforts by the South Coast Air Quality Management District (SCAQMD) to provide an attainment plan for PM_2.5_ exceptionally challenging (*64*).

It is a poor twist of fate that one of the dominant PM components in the LA atmosphere is also one of the most difficult to measure. Data collected by SCAQMD demonstrate that in Southern California, while the Pasadena location attains the 2012 NAAQS, Downtown LA, Pico Rivera, and Rubidoux fluctuate in and out of compliance over the past decade. In aggregate, LA County does not attain this standard (see fig. S16). Correcting the SCAQMD observations to account for AN evaporation (only possible at the sites where an independent measurement of nitrate is made, in this case Downtown LA and Rubidoux), we find that the annual average PM_2.5_ levels at these sites clearly do not meet the 2012 NAAQS, much less the more stringent 2024 requirement (see fig. S17). In a place like LA, knowledge of the PM speciation and the ability to correct for these artifacts is critical, although having correct, unbiased data in the first place is preferable. These observations suggest that the EPA’s FRMs and FEMs are incapable of capturing the true PM_2.5_ mass. We note additionally that PM_2.5_ is a blunt instrument for assessing the concentration of PM that has allowed these biases to persist. While PM_2.5_ has played an integral regulatory role in reducing fine particle levels, it may be time to revisit the emphasis on PM_2.5_ mass and incorporate a contemporary understanding of the complexities of the PM into our regulatory frameworks (e.g., their size distribution or speciation).

**Fig. 6.**
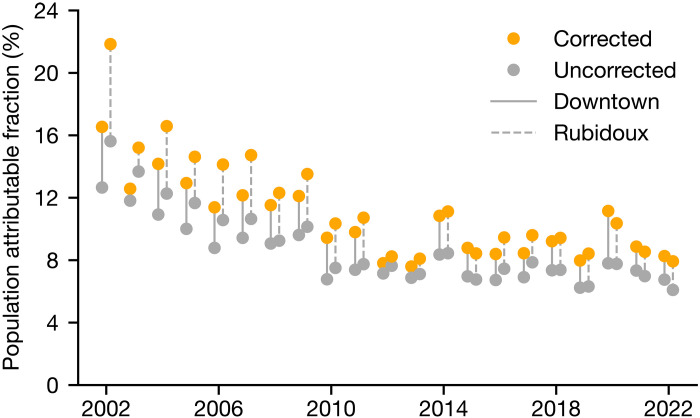
Attributable PM-related health outcomes with and without accounting for AN. Time series of the PAF (multiplied by 100%) for all-cause premature mortality due to long-term (annual average) PM_2.5_ exposure calculated with uncorrected concentrations and concentrations corrected for unobserved AN at the Downtown LA and Rubidoux sites.

Hence, we advocate caution in interpreting the absolute FRM PM data and its trends in regions where AN is high, such as in California. One natural case that arises is in the application of PM data to epidemiological studies. We tested how this pathology influences our understanding of public health outcomes, with a thorough description provided in the Supplementary Materials (section S5). Briefly, we calculated the population attributable fraction (PAF) of total (all-cause) premature deaths in the population attributable to long-term exposure to PM_2.5_ (*65*) with both uncorrected and corrected PM values in Downtown LA and Rubidoux. The results, shown in Fig. 6, suggest a consistently low bias in PAF at both locations; a difference of even just 3 to 4% mapped onto the LA metro population is tens of thousands of people. This difference is likely an underestimate as we account for only the evaporation of AN and not the potential evaporation of other semivolatile species, such as the organic aerosol (*66*). A number of studies use PM_2.5_ data measured via FRMs to generate datasets [e.g., (*56*, *67*)], and in turn these data are central to health effect studies, so the health damages associated with PM_2.5_ are likely to be underestimated in those regions where AN is pervasive (*68*, *69*).

Despite the many strides and advances in our understanding of air quality in LA, AN aerosol remains a top contributor to PM_2.5_ mass. A true measurement of its mass remains elusive, plaguing our understanding of the atmospheric chemistry and public health implications of PM in LA, and certainly many other cities. It is critical that the EPA’s codified techniques for measuring PM are consistent with the state-of-the-science and that they reflect the contemporary understanding of urban air quality.

## MATERIALS AND METHODS

Descriptions of the ACSM measurements in Pasadena and Pico Rivera, the AMS measurements in Pasadena, the instrument correction procedures, the nitrate trend calculations, and the health impacts study can be found in the Supplementary Materials.

## Supplementary Materials

### This PDF file includes:

Supplementary Text Figs. S1 to S23 References
