# Supplementary material for: Poorly quantified trends in ammonium nitrate remain critical to understand future urban aerosol control strategies
Source: Sci Adv. 2025 May 21;11(21):eadt8957. doi: 10.1126/sciadv.adt8957 (PMC12094242; doi:10.1126/sciadv.adt8957)
Supplement: Supplementary file 1 — Supplementary Text Figs. S1 to S23 References [file sciadv.adt8957_sm.pdf]

Science Advances
AAAS

Supplementary Materials for

Poorly quantified trends in ammonium nitrate remain critical to understand future urban aerosol control strategies

Ryan X. Ward *et al.*

Corresponding author: Ryan X. Ward, ryan@caltech.edu

Sci. Adv. **11**, eadt8957 (2025)
DOI: 10.1126/sciadv.adt8957

This PDF file includes:

Supplementary Text
Figs. S1 to S23
References

Supplementary Text

1 Ambient measurements

1.1 Pasadena 2023 (Spring and Summer)

Ambient measurements of non-refractory aerosol chemical composition were made by an Aerodyne Aerosol Chemical Speciation Monitor (ACSM) with a $PM_{2.5}$ inlet from 30 March to 14 June 2023 in Pasadena, CA from the roof of Caltech Hall (~40 m above ground level) (*41*). This contrasts previous aerosol mass spectral measurements in Pasadena, e.g. (*5*), that reported the non-refractory PM_1 with an Aeordyne HR-ToF-AMS. Aerosol was sampled through 7 m of 3/8" stainless steel line attached to a Teflon-coated cyclone mounted to the roof of Caltech Hall. The cut size was set to 3.5 μm (recall this ACSM is a $PM_{2.5}$ measurement, and a typical 2.5 μm cut would serve to doubly-cut the aerosol). The sample is dried by a Nafion dryer to an RH below 15%. Data were analyzed in Tofware and the composition dependent collection efficiency (CDCE) was applied (*70*).

Throughout the campaign, ammonium is roughly balanced by the measured anions (sulfate, nitrate, and chloride; see Figure S18), suggesting little nitrate in the form of organic nitrates.

The PM_1 volume distribution was measured by a Scanning Mobility Particle Sizer (TSI, Classifer Model 3082, water CPC Model 3789). The SMPS mass is inferred from the volume through estimation of the aerosol density (*71*):

$$\rho_{aer} = \frac{SO_4 + NH_4 + NO_3 + Chl + Org}{(SO_4 + NH_4 + NO_3 + Chl)/1.75 + Org/\rho_{org}} \quad (S1)$$

where SO_4, NO_3, NH_4, Chl, and Org are the measured concentrations of the ACSM, 1.75 is the conversion for the density of inorganic constituents, and ρ_{org} is the density of the organic aerosol inferred from the O:C and H:C ratios (*72, 73*). We note that Hu and colleagues (*71*) further correct the aerosol organic density above 1.6 g cm^{-3}, which may bias our SMPS-inferred mass a bit high in this study, though this difference in the organic density is often less than 1% that post-correction, which represents an even smaller difference to the total aerosol difference.

$PM_{2.5}$ is also measured at Caltech Hall using a Teledyne T640 monitor, a Federal Equivalence Method (FEM). This measurement is optical, using Lorenz-Mie theory to convert scattered light into particle loading. When the ambient RH is above 35%, the sample air is dried through heating to

35%, and these temperatures reported by the instrument. Measurements of $PM_{2.5}$ are also made by a PurpleAir PA-II monitor. This too is an optical measurement, inferring mass concentration from scattered light (*74*). Data were accessed through PurpleAir's API. The Plantower ATM algorithm output (for outdoor data) are used.

Additional trace gas and meteorological data are provided by an SCAQMD monitoring station in Pasadena on Caltech's campus, roughly 500 m away from Caltech Hall. These data include: wind speed, wind direction, relative humidity, temperature, CO, NO, NO_2, O_3, and $PM_{2.5}$ (FRM). Data were accessed through the Air Quality and Meteorological Information System (AQMIS).

1.2 Pico Rivera 2023 (Summer and Fall)

Similar measurements to Pasadena were made in Pico Rivera, about 16 km south of Pasadena. While it is comparable to Pasadena in that it is a downwind urban plume from the Los Angeles urban core, it contrasts Pasadena in its proximity to industrial activities and its distance from the foothills of the San Gabriel Mountains (adding meteorological differences). This site is part of the ASCENT project (Atmospheric Science and Chemistry mEasurement NeTwork) and is an SCAQMD monitoring site.

Ambient air was sampled through a cyclone to the ACSM and SMPS via 1/4" stainless steel tubing through a cyclone at a total flow rate of 2 lpm (0.1 lpm for the ACSM, 0.6 lpm to the SMPS, and 1.3 lpm make-up flow). We note the cyclone cutoff diameter is 3.5 μm, as described in the previous section. Calibration and data analysis procedures are the same as previously provided.

Again, SCAQMD provides the following QA/QC measurements: FRM $PM_{2.5}$, O_3, NO_x, and meteorological data (temperature, relative humidity, and wind speed/direction). Additional aerosol measurements include: an aethelometer (AE33, Magee), a metals monitor (SCI Xact 625i), and the SMPS. Additionally, PurpleAir PA-II sensors are deployed at the site.

1.3 Pasadena 2021 (Summer) – RECAP

In the summer of 2021, ambient measurements were made as part of the RECAP campaign in Pasadena by multiple stakeholders, including Caltech, NOAA, EPA, CARB, UC Davis, and others. We are interested in two sets of measurements from this campaign in addition to the aerosol: gas-

phase nitric acid and ammonia. Ammonia (NH_3) was measured by CARB's Picarro SI2103 Gas Concentration analyzer. Nitric acid (HNO_3), among other species, was measured via two different Chemical Ionization Mass Spectrometers (CIMS) operated by Caltech and NOAA, respectively. Briefly, these two measurements use differing reagent ions, CF_3O^- in the case of Caltech and I^- in the case of NOAA (*75, 76*). HNO_3 is a notoriously difficult species to measure for a variety of reasons; in particular here, we focus on condensed-phase interferences, which have been known historically to affect HNO_3 measurements from filter techniques, though, to our knowledge, not yet published for CIMS techniques from these specific instruments (*24, 63, 77*).

During the campaign, the Caltech and NOAA measurements report substantially different measurements of HNO_3, shown in Figure S15 as the residual of the two instruments (the raw HNO_3 measurements are also scattered in Figure S19 for clarity). A high bias is observed in the NOAA CIMS relative to the Caltech CIMS, which we hypothesize as interference from volatilization of condensed-phase nitrate.

While speciated $PM_{2.5}$ was not measured, we do have speciated PM measurements from the HR-ToF-AMS (instrument operation details as in Section 1.4), and we can infer the $PM_{2.5}$ composition as follows: (1) we take the total mass of $PM_{2.5}$ from the PurpleAir sensor at the ground site and (2) we infer the composition of the $PM_{1-2.5}$ as either uniform to the PM_1 or entirely enriched in ammonium nitrate. To the first point, we believe the total mass of $PM_{2.5}$ is (to good approximation) represented by the PurpleAir sensor, as pointed out in the Main Text. To the second point, we also believe that both of these cases are possible given previous literature (e.g., (*48*)) and our results in the Main Text. So, the uniform $PM_{1-2.5}$ composition with the PM_1 represents likely a lower bound on the amount of ammonium nitrate present in the $PM_{2.5}$, and the enriched case represents the upper bound.

With this in mind, Figure S15 demonstrates that if evaporated particulate nitrate drives the differences in the two CIMS measurements, there is likely sufficient particulate nitrate present on any given day to explain the difference. A likely explanation for the high bias of one of the CIMS is that a long and warm inlet led to the evaporation of ammonium nitrate aerosol, yielding much higher HNO_3. Furthermore, the timing of the differences (e.g., largest differences at night) is consistent with the nighttime formation mechanism for ammonium nitrate.

1.4 Pasadena 2022 (Summer) – LAAQC and CalNexT

In the spring and summer of 2022, a series of AMS (PM_1) and CIMS measurements were made in Pasadena, CA at Caltech Hall, the same location as in our ACSM measurements a year later. These campaigns were termed LAAQC (15 May - 23 June) and CalNexT (2 July - 14 August). AMS measurements were made though the same inlets as described for the 2023 Pasadena measurements (Section 1.1). Differently, ambient air was dried via a 24” Nafion dryer and the air was sampled every 5-minutes (the other 5-minutes the AMS was sampling an oxidative flow reactor). Data were analyzed using the SQUIRREL v1.65C and PIKA v1.25C packages, applying the composition-dependent collection efficiency and the “improved ambient” elemental analysis (*70, 72*). Data from these campaigns are presented in Figure S4.

VOC measurements were made via whole air sampling, described elsewhere (*37, 38*). These VOC measurements from the afternoon (2-3 PM) were used to estimate the air mass OH exposure (*78*). These data, among other campaigns, are presented in Figure S20.

To calculate the speciated $PM_{2.5}$, we follow the same protocol as in the 2021 AMS dataset, employing the PurpleAir to get the magnitude of the total mass of the $PM_{2.5}$ and using the AMS and assumptions about the $PM_{1-2.5}$ to tease out the amount of secondary nitrate.

2 Instrument Corrections

2.1 Federal Reference Method

To correct the Federal Reference Method data at various SCAQMD sites, we used a parameterization proposed by Hering and Cass, adapted recently by Chiu and Carlton, herein briefly described (*32, 35*). Three mechanisms for inducing loss are considered: (1) the depletion of ammonia and nitric acid ahead of the filter, (2) an increase in temperature of the filter above ambient, and (3) pressure drop across the filter. Using the dissociation constant for ammonium nitrate (K_{amb}), the loss is calculated as:

$$\Delta NO_3^- = \frac{745.7}{T_R}\frac{1}{24}\sum_{i=1}^{24}\sqrt{\ _{amb}} \quad \text{(S2)}$$

where ΔNO_3^- is the loss of nitrate in [μg m^{-3}], T_R is a reference temperature, and 745.7 results from unit conversions. For details of the calculation of the dissociation constant and other relevant

thermodynamic quantities (such as the deliquescence relative humidity), we refer the reader to the above citations.

Important to note, this calculation represents an asymptotic loss rate of nitrate, while the real situation is time-dependent. In other words, the depletion of nitric acid or ammonia above the headspace of the filter, in addition to the temperature of the filter, are time-variant quantities, so the evaporation rate of ammonium nitrate (or condensation, if applicable) is a function of time. Here, the loss rate is bounded by true measurements of the particulate nitrate; in the work of Hering & Cass a denuded nylon filter is used to know the true value, and in this work, we employ both the nylon filter and the ACSM to bound the measurements. With known amounts of nitrate, we ensure a physical upper limit to the volatilization of nitrate.

2.2 Federal Equivalence Method

To correct the data for the Federal Equivalence Method, in this case the Teledyne T640 data, it is useful first to understand its operating principles. In brief, to make its measurement optically, and to ensure comparability of the aerosol population in various humidities, the instrument heats the sample to achieve a relative humidity of 35% (we note that the ACSM uses a Nafion dryer, which can ostensibly introduce artifacts as well (*66*)). This heating could drive volatilization of the semi-volatile species in the sampl, so we employed the thermodynamic model ISORROPIA-II to correct for this artifact (*79*).

Briefly, ISORROPIA-II is a thermodynamic equilibrium model which solves for the equilibrium state of various species, including ammonium, nitrate, and sulfate. There are two modes of operation: forward and reverse. In the forward mode, the equilibrium state is calculated for known gas and aerosol phase species plus temperature and relative humidity. In the reverse mode, only condensed phase concentrations are known, and the model outputs the gas, liquid, and solid phases. This latter mode is often useful in interpreting ambient observations where often only condensed phase values are known. We also note errors in the reverse mode, principally with respect to pH (*80*), though we do not expect this pathology to impact the results here.

To start, we run the model in reverse mode for the following conditions: the ACSM-measured sulfate, nitrate, ammonium, chloride, and the temperature and relative humidity measured at the

TCCON station on Caltech's campus (roughly 100 m away). Phase state was set to metastable and crustal species were ignored. With this information, the aerosol and gas-phase species concentrations were estimated.

The Teledyne instrument records the temperature and relative humidity of the sample in real-time, which may differ from the ambient temperature/RH due to the sample heating described above. We use these measurements to correct for the temperature-driven volatilization of ammonium nitrate under the assumption that the sample arrives at thermodynamic equilibrium. This is not a terrible assumption, as we know the ammonium nitrate system can reach thermodynamic equilibrium on the order of minutes depending on the distributions of size and phase state (*81*). Using the results of the reverse run of ISORROPIA as a starting point, we run the model in forward mode using the total calculated aerosol and gas nitrate, sulfate, ammonium, and chloride; however, we use the sample temperature and relative humidity reported by the instrument. In essence, our goal is to re-partition these inorganic constituents between the aerosol and gas phases at the elevated temperature reported by the instrument. From here, we extracted the expected aerosol-phase concentrations of the nitrate, sulfate, and ammonium, and we adjusted the ACSM concentrations to match these values, thereby allowing the ACSM measurement to reflect any temperature-driven volitilization that the Teledyne instrument would experience. The results of the correction are displayed in the Main Text (Figure 5(a) and (d)) and reproduced in Figure S13(a) and (b).

Sensitivity studies were performed to test the efficacy of the reverse mode of ISORROPIA-II. We inflated the gas-phase concentrations of nitrate (HNO_3) and ammonia by 2 and 10x the output of the initial reverse mode simulations to test how sensitive the re-partitioning is to the gas-phase concentrations (which are unmeasured). For a greater gas-phase concentration of either species, we would expect the aerosol-phase to retain more of the species, thereby limiting the artifact induced by heating of ammonium nitrate. These tests are shown in S13(c) and (d). We observe in either case that even if the reverse mode of ISORROPIA-II initially output gas concentrations that are too low, by inflating that gas-phase concentration we can still create good agreement just by repartitioning ammonium nitrate. Primarily, we are interested in the plausibility of ammonium nitrate partitioning in explaining the differences between the ACSM and Teledyne instruments, and while quantitatively this correction is quite accurate, we acknowledge that this technique is mechanistically qualitative, as we never actually constrain the amount of ammonium nitrate in this instrument.

2.3 EPA Corrections to the Teledyne T640/X (FEM)

In May of 2024, the US EPA issued guidance under 40 CFR 53.14 to retroactively adjust the data output by Teledyne T640/X monitors; all future data published using this FEM would be amended with this modification (*53*). The purpose of this correction is to improve a consistent positive bias observed between the T640/T640X instruments and the Federal Reference Method. The report from Teledyne further details the positive bias to be larger at cooler temperatures. This calibration applies a temperature correction to the Teledyne data as follows:

$$\mathrm{PM_{corr}} = \begin{cases} 0.813233 \times \mathrm{PM_{meas}} & \mathrm{T} < 20, \mathrm{PM_{meas}} \leq 10 \\ \mathrm{PM_{meas}} - 1.861 & \mathrm{T} < 20, \mathrm{PM_{meas}} > 10 \\ 0.813233 \times \mathrm{PM_{meas}} & \mathrm{T} > 20, \mathrm{PM_{meas}} \leq 5 \\ \mathrm{PM_{meas}} - 0.925 & \mathrm{T} > 20, \mathrm{PM_{meas}} > 5 \end{cases}$$

where $\mathrm{PM_{meas}}$ is the output concentration by the T640/X [μg m^{-3}], T is the temperature [°C], and $\mathrm{PM_{corr}}$ is the corrected $\mathrm{PM_{2.5}}$ value to report [μg m^{-3}]. Uniformly, this data correction adjusts down the Teledyne data concentration, which is perhaps antithetical to the biases reported in this work. The results of the correction applied to the 2023 Pasadena dataset are shown in S13(e) and (f), where we compared the adjusted values to the ACSM and the FRM. In both cases, the Teledyne underestimates the reported concentration, further generating a negative bias of the instrument performance, as expected based on the operating principles of the instrument and the thermodynamics of the aerosol population.

3 Trends calculations

3.1 Nighttime Nitrate Radical Production

In general, the production of nitrate radical is governed by one reaction:

$$O_3(g) + NO_2(g) \rightarrow NO_3(g) + O_2(g) \qquad k = 1.2 \times 10^{-13} \exp\left(-\frac{2450}{T}\right). \tag{S3}$$

This is the only source of nitrate radicals in the nighttime urban atmosphere, where it is the dominant radical initiating chemistry. During the day, NO_3 is photolyzed and lost, so its chemistry

is not considered (though aerosol-phase inorganic nitrate can be produced via the OH-initiated oxidation of NO_2).

We estimate the production rate of nitrate radicals via the relationship

$$P(\mathrm{NO_3}) = k(T)[\mathrm{NO_2}][\mathrm{O_3}], \tag{S4}$$

which gives the instantaneous production rate based on measured NO_2 and O_3 concentrations and the temperature. We sum this production rate over the evening (determined by the measured solar irradiance at Caltech's TCCON station) to estimate the total production of NO_3 radicals. Ultimately, the conversion of NO_3 radicals to aerosol phase NO_3^- is governed by the efficiency of the following reactions:

$$\mathrm{NO_3}(g) + \mathrm{NO_2}(g) \rightarrow \mathrm{N_2O_5}(g), \tag{S5}$$

$$\mathrm{N_2O_5}(g) + \mathrm{M}(g) \xrightarrow{\Delta} \mathrm{NO_3}(g) + \mathrm{NO_2}(g) + \mathrm{M}(g), \tag{S6}$$

$$\mathrm{N_2O_5}(g) + \mathrm{H_2O}(aq) \rightarrow 2\mathrm{HNO_3}(aq), \tag{S7}$$

$$\mathrm{HNO_3}(aq) + \mathrm{H_2O}(aq) \leftrightarrow \mathrm{NO_3^-}(aq) + \mathrm{H_3O^+}(aq), \tag{S8}$$

$$\mathrm{NO_3^-}(aq) + \mathrm{NH_4^+}(aq) \leftrightarrow \mathrm{NH_4NO_3}(aq). \tag{S9}$$

If we assume (1) the conversion of nitrate radical to aerosol nitrate was local (i.e., it is sufficiently rapid that the appearance of the particle phase nitrate corresponding to changes in NO_2 and O_3 can be observed at the same location), and (2) that the particle-phase nitrate is in turn not transported away, then we can estimate the production of particle-phase nitrate solely from NO_2 and O_3. In essence, we want to know how efficiently every molecule of NO_2, if following the reactions (S3) and (S5)-(S9), is converted to particle phase NO_3^-.

These calculations are depicted in the Main Text in Figures 2 and 4, and reproduced and amended with further data in Figures S8 and S9. From trace gas measurements, we sum the cumulative nighttime production of nitrate radical from NO_2 and convert this into particle-phase nitrate through the above chemical reactions, Then, we compare this to the observed amount of nitrate formed overnight measured by the ACSM or AMS, the latter of which has been adjusted to reflect $PM_{2.5}$ (as previously described). For each night, a measurement that falls close to the 1:1 line (which we have called the "local chemical production limit") suggests that the NO_2 is efficiently converted to particle-phase nitrate in the absence of transport.

For springtime and early summer (as in Main Text Figure 4(a)), we see much of the data clusters at the local chemical production limit, which suggests that reductions in NO_x may serve as an effective control strategy for particulate nitrate, a similar result to other locations and seasons (*23, 82*). Across this time period we see that the primary constraint on particle-phase nitrate formation is water, suggesting a limited hydrolysis of N_2O_5. In contrast, in the summer (Pico Rivera and CalNexT) we see fewer days which tend towards this local chemical production limit, which potentially suggests limitations through other mechanisms, for instance NH_3, or simply temperature, shown by Figure S9. Indeed, a variety of chemical and physical constraints to ammonium nitrate formation have been documented, so its control can not only be left up to NO_x mitigation strategies (*15, 83*). We discuss this further in Supplemental Section 4.

3.2 Daytime Nitric Acid Production Proxy

To estimate the production of daytime gas-phase nitric acid, we are roughly seeking to understand this reaction:

$$NO_2(g) + OH(g) \rightarrow HNO_3(g). \tag{S10}$$

While direct measurements of OH are difficult to make, a proxy for OH, the OH exposure (*78*), has been measured numerous times in LA, and we leverage this to calculate the trend in nitric acid production (*5, 37*). During LAAQC and CalNexT measurements of NO and midday OH exposure (inferred from VOC measurements) were measured in Pasadena, and in comparison with observations in 2010 from CalNex, they largely suggest OH exposure has increased over the last decade and track NO. These data are shown in Figure S20. We fit this data with an exponential fit of the form:

$$OH_{\mathrm{exp}} = a\exp(b[\mathrm{NO}]), \tag{S11}$$

where OH_{exp} is the afternoon OH exposure measured between 2 and 3 pm, NO is the mean afternoon NO concentration (1 to 5 pm average), and a and b are fit parameters. We use this fit to infer OH_{exp} on a decadal timescale, using mean afternoon NO values from Pasadena recorded between 1994 and 2022 (implicitly assuming that despite changes in the relative composition of the LA atmosphere, and in particular the VOC composition, that changes in NO can sufficiently track changes in OH exposure). Other work (derived from remote sensing measurements) has suggested

an increase in OH in the basin over similar timescales (*84*) (consistent with declining NO_x, as in this parameterization). With the OH_{exp}, we can then infer a proxy for total daytime nitric acid production, which we take to be the product:

$$P(HNO_3) = k(T)OH_{exp}\overline{[NO_2]}, \tag{S12}$$

where OH_{exp} is the mean daytime OH exposure (inferred at 2 to 3 pm), $\overline{[NO_2]}$ is the mean afternoon NO_2, and $k(T)$ is the mean daytime rate constant for the reaction. Since OH exposure is the integrated exposure of the air mass around mid-afternoon, we multiply this value by the fraction of day remaining to approximate a full integrated day of oxidative aging (e.g., if sunrise were at 6 am, OH_{exp} measured at 2 pm, and sunset at 10 pm, we would multiply this product by 2, since we've only measured half of the integrated exposure to OH). Assuming NO_2 is relatively constant in the air mass over the day, we generate a proxy for the total cumulative production of HNO_3 in the air mass. Implicitly, this reaction suggests a competition between declines in NO_x in the basin and increases in OH vis-à-vis the production of HNO_3.

With a knowledge of the decadal changes in the production of nitric acid and nitrate radical, which are the presumed dominant contributors to day and nighttime nitrate aerosol respectively, then we can, at a high level, understand changes in the relative importance of day and nighttime formation processes in nitrate aerosol.

4 Thermodynamics of Nitrate Partitioning

Reductions in either (or both) NO_x and NH_3 can act as levers for reducing the atmospheric burden of AN. So, it is useful to identify which of these (if either) is the limiting reagent, so it can be targeted for control. For the LA basin in 2010, it has been suggested that the AN is sensitive both to total NH_3 and total NO_3 burdens, meaning that reducing emission of either of these precursor species can serve as an effective strategy for reducing the aerosol burden (*26*). The two limitations (by NO_x or by NH_3) are summarized well by Guo and coauthors (*25*). Briefly:

1. *NO_x Control.* Reduction in the precursor of HNO_3 formation, namely NO_x, should reduce the burden of AN aerosol by removing nitrate from the system. This is only effective if a

majority of the total NO_3 is partitioned to the particle phase, which happens under conditions of less acidic aerosol and higher aerosol liquid water content.

2. *NH_3 Control.* Reduction in NH_3 concentrations primarily impacts particle pH, increasing the acidity. This shifts the partitioning equilibrium of the nitrate out of the aerosol-phase and into the gas-phase, thereby reducing AN. If particle pH is sufficiently high and all sulfate neutralized, reductions in NH_3 may not substantially impact the particle-phase concentration of nitrate, as it will likely already reside in the aerosol phase.

Since 2010 in Los Angeles, NO_x concentrations and emissions have declined while NH_3 emissions have remained largely stagnant (Figures S1 and S2). The trend in NH_3 concentrations is less well-constrained; measurements in late spring/early summer of 2010 in Pasadena suggest an average daily NH_3 concentration of 2 μg m^{-3}, whereas measurements in late summer of 2021 suggest somewhere around 5 μg m^{-3} (Figure S10). Because of the meteorological and seasonal impacts on emissions and atmospheric concentrations, it is tough to say that the ambient concentration is increasing on those two data points; however, it is likely not decreasing, a trend consistent with measurements in many parts of the world (*29, 30*). So, with declines in NO_x and not NH_3 it stands to reason that AN formation may be trending towards a NO_x limitation judged on atmospheric abundance alone. This of course misses the complicated interplay between pH (modulated by aerosol sulfate), aerosol liquid water, temperature, and other factors.

To examine these limitations more rigorously, we assessed the partitioning of nitrate for measurements made during the RECAP campaign (Pasadena Summer 2021; measurement description in Section 1.3). While the composition of the $PM_{2.5}$ was not measured, as discussed, we try to infer the dynamics of the nitrate partitioning to generalize about the trends in nitrate production over the last decade. To determine the partitioning, ISORROPPIA-II (model decsription in Section 2.2) was run in forward mode using the following inputs: the $PM_{2.5}$ speciated mass is assumed to be that of the enriched case described in Section 1.3, the HNO_3 concentration is taken from the CIT CIMS, and the NH_3 concentration is taken from the Picarro measurements. Crustal species are ignored. To test limitation by either NO_x or NH_3, we run five simulations variations: a base case (all concentrations as measured), two where the total NH_3 and total NO_3 (gas and particle phase sum) are independently reduced by 50%, and two where they are reduced by 90%.

The results of these simulations are shown in Figure S11. Akin to the finding of Guo and colleagues (*25*) in the Netherlands, we find that for the summer of 2021, LA exhibits a stronger initial sensitivity to reductions in total NO_3, suggesting that controls on NO_x remain an important control strategy for PM. For a large reduction in total NH_3, there is a strong enough shift in the pH to influence the nitrate partitioning (away from the particle-phase), though the reduction in PM is likely more modest at lower total NH_3 reductions. Furthermore, following Nenes et al. 2020 (*26*), we identified that, for much of the campaign, predicted aerosol pH and aerosol liquid water content suggest that the air masses are more sensitive to HNO_3, and therefore NO_x. The results are shown in Figure S12. This contrasts the results for 2010 (shown in the Nenes reference, Figure 5(b)), where the majority of the data lie in a regime sensitive to both NH_3 and NO_x, though it is consistent with our understanding of the trends in aerosol sulfate and the relative abundances of NO_x and NH_3.

We stress that these results are more qualitative than quantitative, as the $PM_{2.5}$ speciated aerosol measurements are not known. However, the dynamics, and in particular the trend over the last decade, are likely representative.

5 Health Impacts

As detailed in the Main Text, to understand how the use of uncorrected $PM_{2.5}$ concentrations impact estimated public health damages associated with $PM_{2.5}$ exposure, we calculate the population attributable fraction (PAF) with both uncorrected and corrected FRM PM measurements. Here, the PAF represents the proportion of total (all-cause) premature deaths in the population attributable to long-term exposure to $PM_{2.5}$ (*65*). We consider annual average concentrations as surrogates for long-term exposure, and we impose a log-linear relationship on relative risk (RR) estimates, that is:

$$\beta = \ln RR \tag{S13}$$

$$PAF = 1 - \exp\left(-\beta \times PM_{2.5}\right) \tag{S14}$$

where RR = 1.03 (95% CI: 1.01–1.05) per 5 μg m^{-3} $PM_{2.5}$. This RR estimate derives from a recent systematic review and meta-analysis from the Health Effects Institute (*85*), which assessed health impacts associated with long-term exposure to traffic-related pollution. While other RR estimates exist, the traffic-specific nature of our RR estimate is particularly relevant in the urban context of

Los Angeles, where traffic is an important contributor to total $PM_{2.5}$ mass and trends (*22*). We discuss these calculations in the Main Text.

$PM_{2.5}$ mass, rather than number or composition, is currently the regulated quantity in the U.S. and many other parts of the world. However, while all components are associated with elevated mortality (*86*), growing evidence generally supports differential toxicities of individual $PM_{2.5}$ components. Generally, the strongest associations of $PM_{2.5}$ components and mortality are found for fossil fuel combustion (i.e., coal and traffic) components, while crustal and biomass components are generally less strongly associated with mortality (*18, 87, 88*). While understanding differential toxicities of components is still an emerging field, having observations and semi-empirical datasets based on observations that reflect the actual composition is a prerequisite for linking composition with adverse health effects.

6 CPF Analysis

In the absence of strong Santa Ana winds, which flush out the LA basin, we don't expect transport to play a dominant role in nighttime aerosol observations. This contrasts with the daytime, where in Pasadena, for instance, the urban plume arrives in the mid-afternoon due to southwesterly prevailing winds, driving up aerosol concentrations with secondary aerosol. To investigate the hypothesis that secondary nitrates are not primarily driven by longer-range (10s km) transport, we performed a conditional probability function (CPF) analysis on the Pasadena ACSM dataset. In brief, this is a receptor modeling approach which uses joint observations of concentration, wind speed, and wind direction to identify the local and long-range sources of air pollutants (*89*). Concentration data are mapped to wind speed and direction using a Generalized Additive Model (GAM). A threshold for concentration is then set to identify dominant emissions sources at that interval (e.g., a source contributing to the 90th percentile of observed concentrations, like a stack plume from a coal-fired power plant vis-à-vis SO_2 concentrations). These functions are viewed on a radial plot (similar to a wind rose) for visual interpretation.

We target the 90th percentile of nighttime ammonium nitrate concentrations (8.5 μg m^{-3}), which reveals that the footprint of ammonium nitrate production is generally local and independent of wind direction, shown in Figure S21. This footprint suggests that the production of ammonium

nitrate is generally local, so calculating the relationship between the gas-phase precursors and observed aerosol concentrations (as in Figure 4 of the Main Text) is not appreciably confounded by transport of either aerosol or gas-phase species.

7 CEPAM Inventory

The California Emissions Projection Analysis Model (CEPAM2019v1.03) was used to determine relative trends in NO_x and NH_3 emissions (*90*). Data were accessed for LA County in the summer, winter, and annual mean (it is expected equinox seasons will track the annual mean) for the years 2000 to 2023. The results are shown in Figure S1. We observe a robust decline in NO_x emissions over this time span (over 60%), but variable and roughly constant NH_3 emissions. Specifically for NH_3, it could possibly be argued that sub-selected to just the last decade, NH_3 emissions are increasing. While the model outputs in [ton day^{-1}], we have converted to [mol day^{-1}] for ease of comparison on a molecule-by-molecule basis. To do this for NO_x, we have taken two limits to give us bounds on the emission: one where we assume all NO_x is emitted as NO and another where all NO_x is emitted as NO_2. It is apparent that NO_x emissions outnumber NH_3 emissions by roughly a factor of 10 on a mole basis. Post atmospheric transformations, these two species may exist in relatively close abundances on a mole-for-mole basis, and so it is not immediately intuitive that the LA basin should be limited with respect to ammonium nitrate by either one of these precursors.

8 CMAQ EQUATES

EPA's Air QUAlity TimE Series Project (EQUATES) is a series of model runs by the EPA's CMAQ model, generated for the purpose of understanding trends in air quality (*57*). These data are available from their website and were accessed on July 1, 2024. We compared EQUATES output data for Pasadena with FRM data collected at that location, the results shown in Figure S14. Specifically, we sub-selected from spring and early summer (March — June), when we expect ammonium nitrate concentrations to be high, for the years 2014 — 2019.

In general, the model agrees well with the FRM data, and the observed ammonium nitrate mass fractions predicted by CMAQ are significantly lower than observed by the ACSM in 2023. Because

it is likely the FRM is evaporating ammonium nitrate, and given the correlation between the FRM and CMAQ, we expect that CMAQ is under-predicting the amount of AN. Over this period, we calculated the expected and observed production of nitrate aerosol, similar to our calculations with the ACSM and AMS datasets. These results are shown in Figure S22.

While it is clear that RH can play some role in inhibiting or promoting nitrate aerosol, as expected, what is more obvious is that the model almost never converts the available nighttime gas-phase NO_3 reservoir into nitrate aerosol. This underestimated nitrate production has been documented in other chemical transport models, such as GEOS-CHEM (*91*).

9 AQMD Data

From EPA data we retrieve and resample PM and trace gas data from various sites in the South Coast Air Quality Management District (SCAQMD), and we correct these data for ammonium nitrate partitioning as described in the FRM section. Data were accessed through the Air Quality and Meteorological Information System (AQMIS). These are presented in Figures S2, S7, S16, S17, and S23.

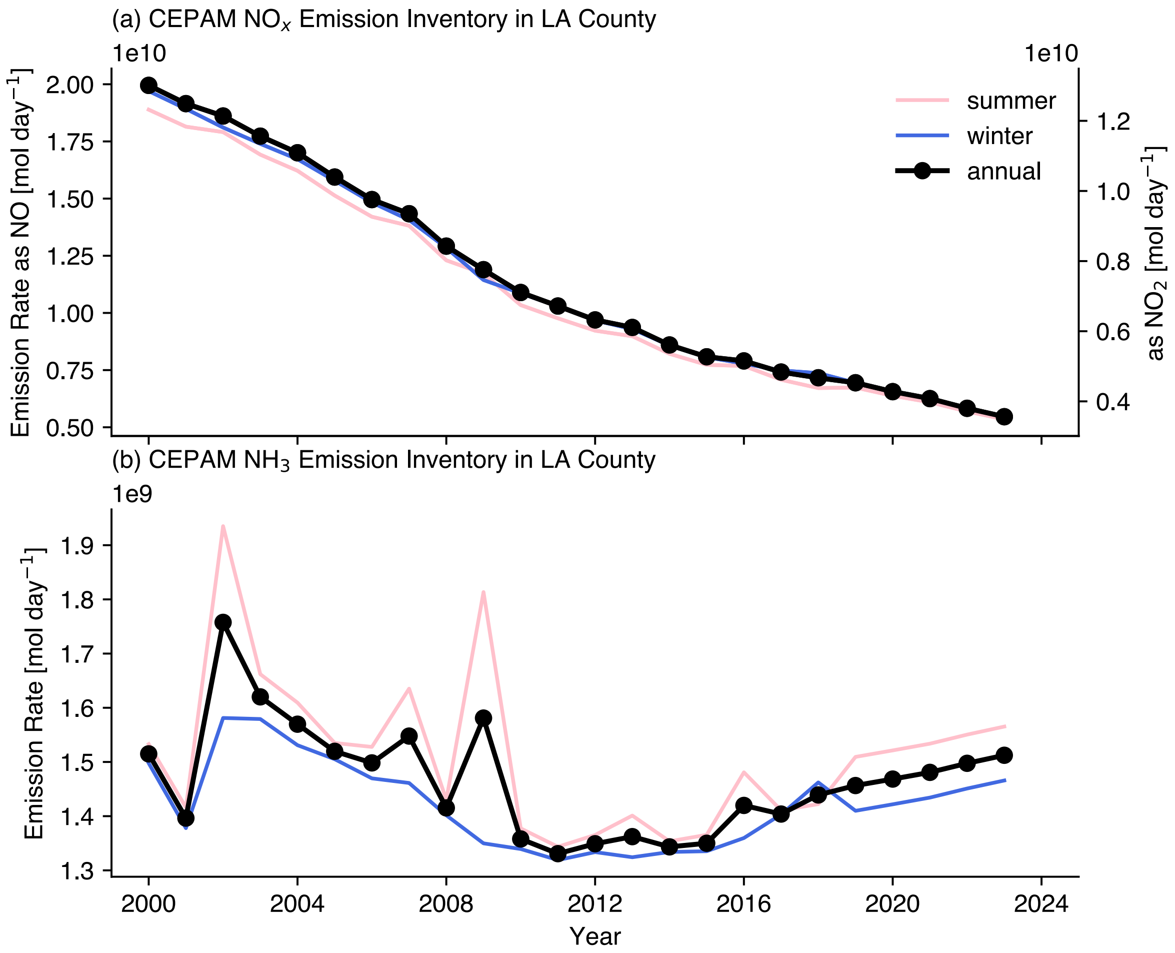

Figure S1: **Time series of NO_x and NH_3 emissions in LA County, provided by the California Emissions Projection Analysis Model (CEPAM2019v1.03).** Emissions are reported by CEPAM in [ton day^{-1}] and here converted to [mol day^{-1}]. For NO_x, we convert to the limit cases of either NO_x emitted all as NO or all as NO_2.

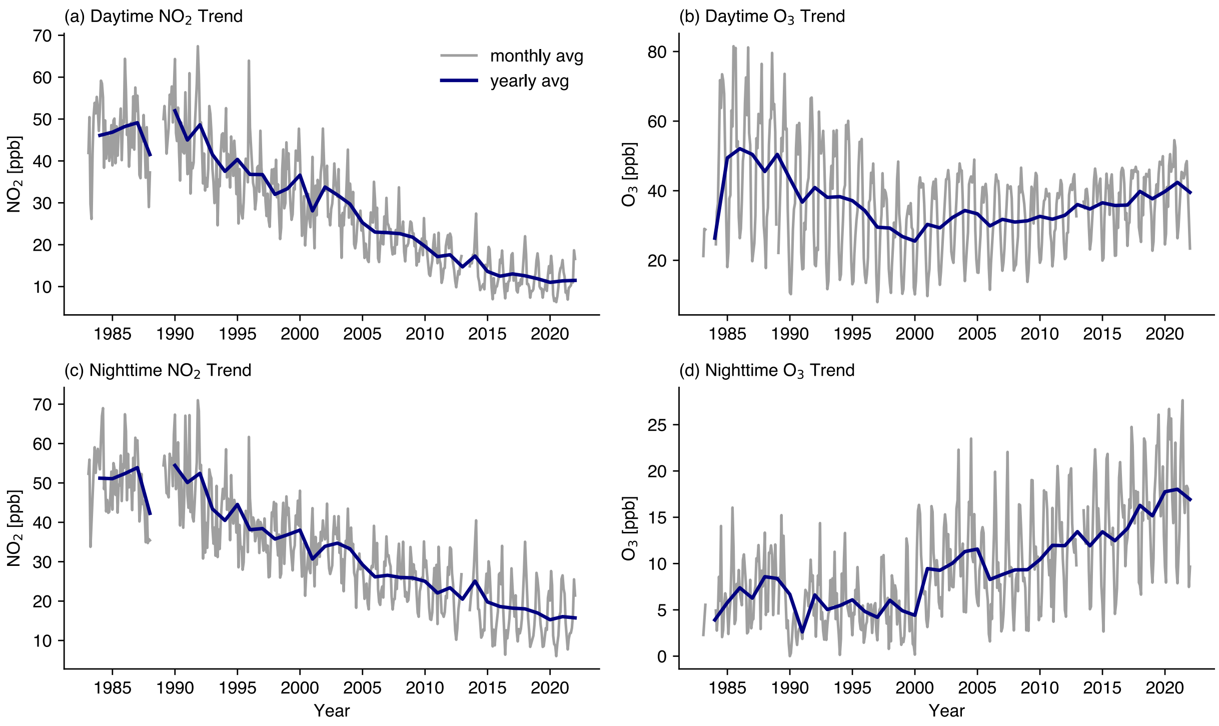

Figure S2: **Time series of NO_2 and O_3 concentrations in Pasadena from SCAQMD**. Panels **(a)-(b)** show daytime values and panels **(c)-(d)** show nighttime values. The grey represents monthly averaging and the blue yearly averaging.

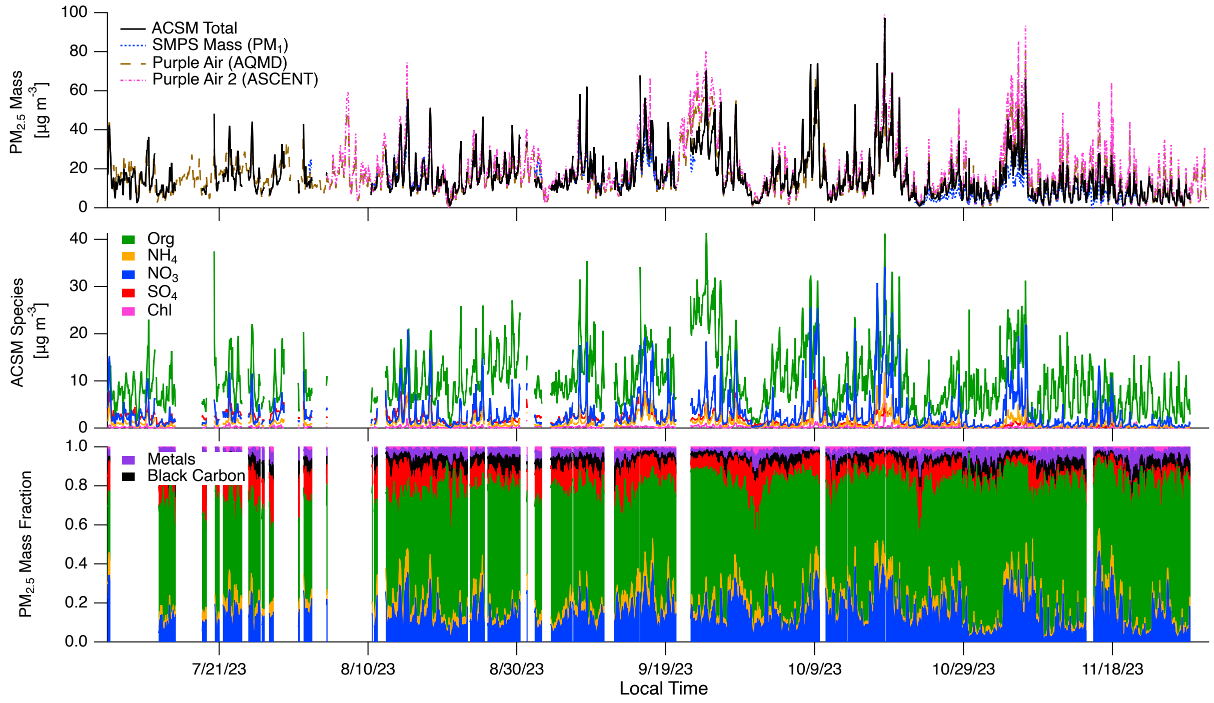

Figure S3: **Pico Rivera campaign data**. Time series of data from various instruments in Pico Rivera during summer and fall of 2023.

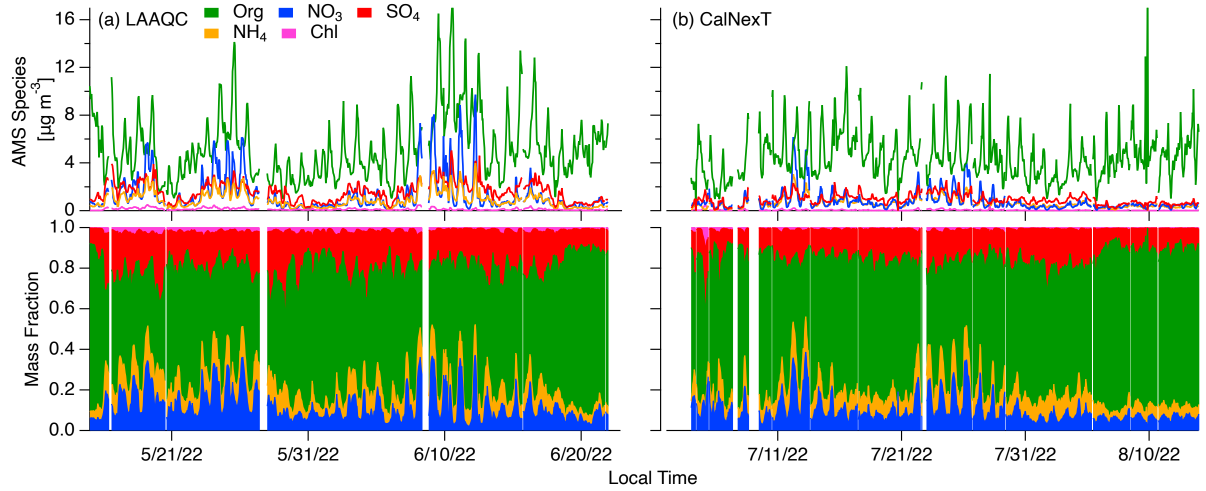

Figure S4: **AMS data from the LAAQC and CalNext campaigns. (a)** LAAQC ran from 15 May to 23 June 2022. **(b)** CalNexT ran from 2 July to 14 August 2022. This AMS had a PM_1 aerodynamic lens.

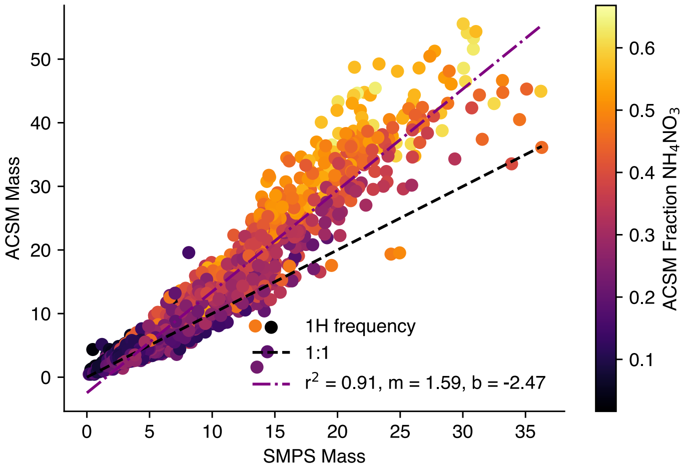

Figure S5: **Scatter plot of time series measurements made by the ACSM and the SMPS in 2023 springtime Pasadena.** SMPS mass is inferred from the aerosol density calculated in Equation S1. Recall, the ACSM is a $PM_{2.5}$ measurement and the SMPS is [approximately] a PM_1 measurement.

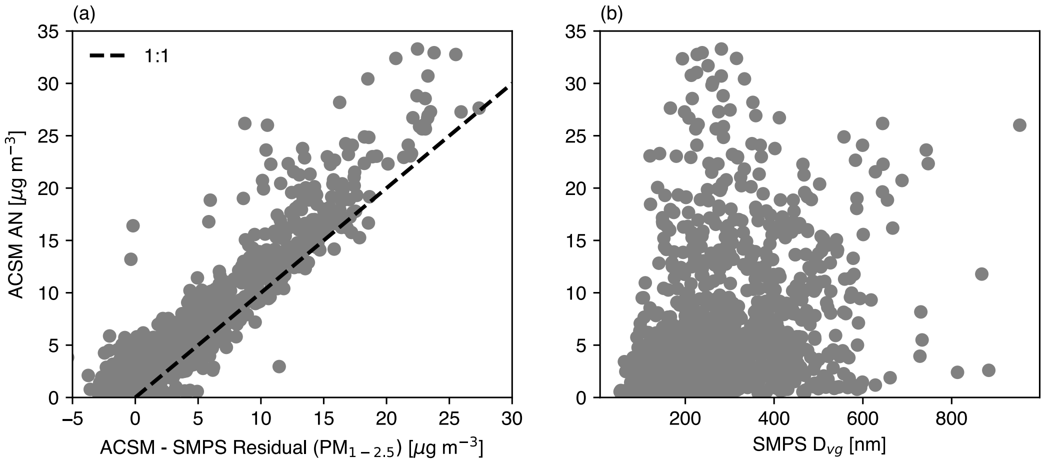

Figure S6: **ACSM and SMPS ammonium nitrate comparisons. (a)** Scatter of the measured ACSM ammonium nitrate (AN) against the residual between the ACSM mass and the inferred SMPS mass, which is essentially the mass $PM_{1-2.5}$. The close correlation suggests much of this mass is ammonium nitrate. **(b)** Scatter plot of the volume median diameter measured by the SMPS and the ACSM ammonium nitrate.

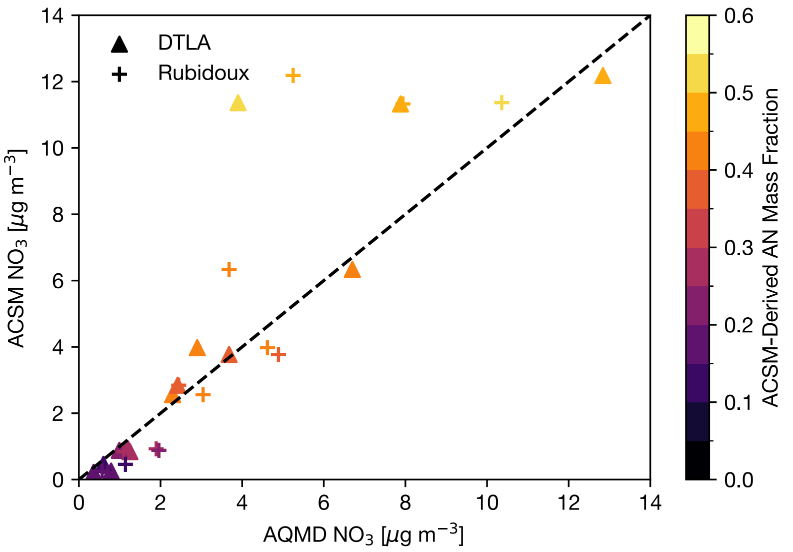

Figure S7: **Comparison between ACSM particulate nitrate measurements in Spring of 2023 with filter measurements made by CSN**. The two locations in Southern California closest to Pasadena and Pico Rivera with speciated PM measurements (by offline liquid chromatography) are Riverside-Rubidoux (shown by the + marker) and Downtown LA (DTLA; shown by the triangle marker). Points are colored by the ACSM-derived ammonium nitrate mass fraction. Each point is a 24-hr average (though days are not consecutive). AQMD is shorthand for Air Quality Management District, in reference to the filter measurements.

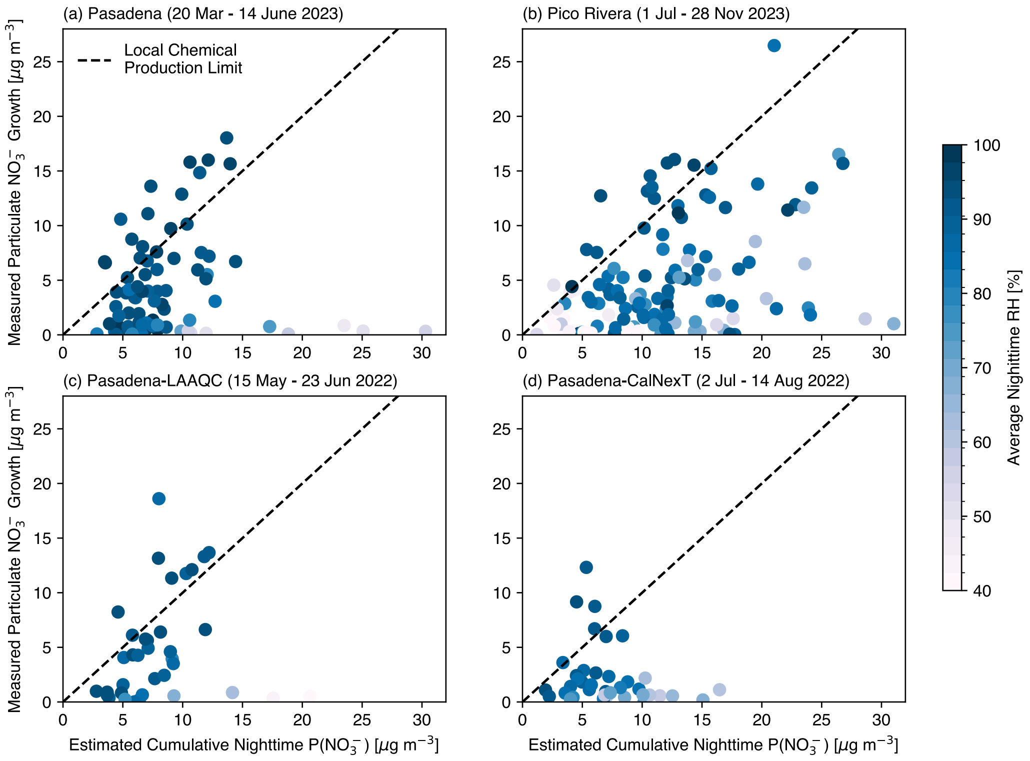

Figure S8: **Estimated and measured production of NO_3^- aerosol**. Calculated for each day in **(a)** 2023 springtime Pasadena, **(b)** 2023 summer/fall Pico Rivera, **(c)** 2022 spring/early summer Pasadena, and **(d)** 2022 late summer Pasadena. Data points represent a single night and are colored by mean RH over the night. Panels (a) and (b) are reprinted from the Main Text.

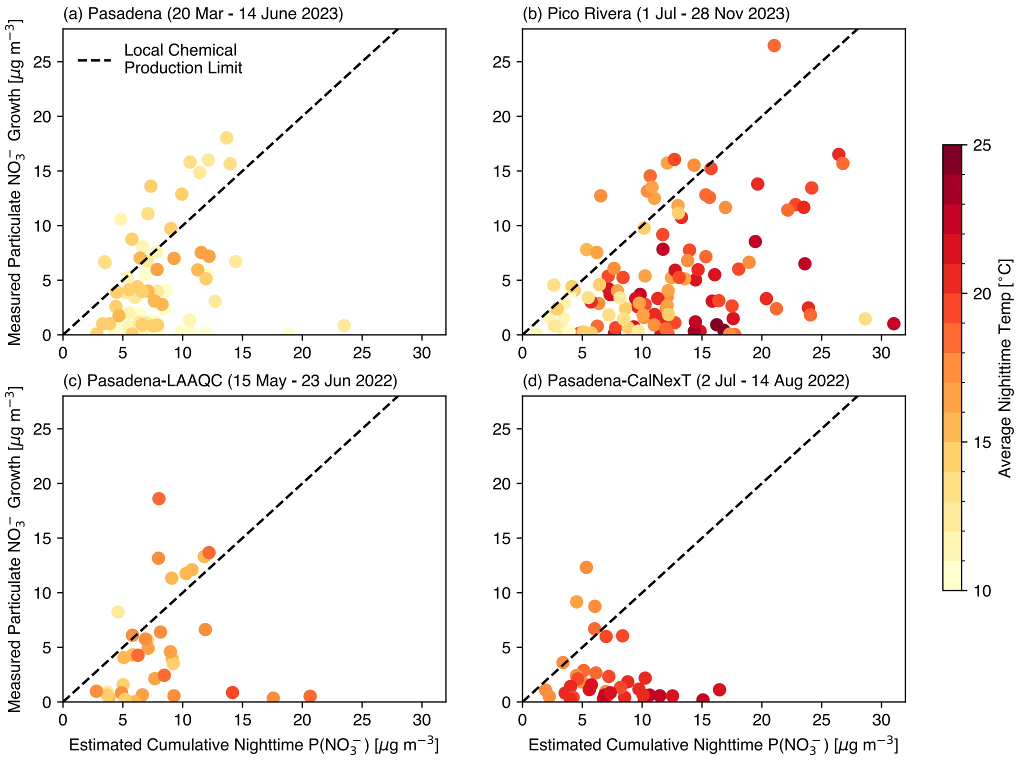

Figure S9: **Estimated and measured production of NO_3^- aerosol**. Calculated for each day in **(a)** 2023 springtime Pasadena, **(b)** 2023 summer/fall Pico Rivera, **(c)** 2022 spring/early summer Pasadena, and **(d)** 2022 late summer Pasadena. Data points represent a single night and are colored by mean temperature over the night.

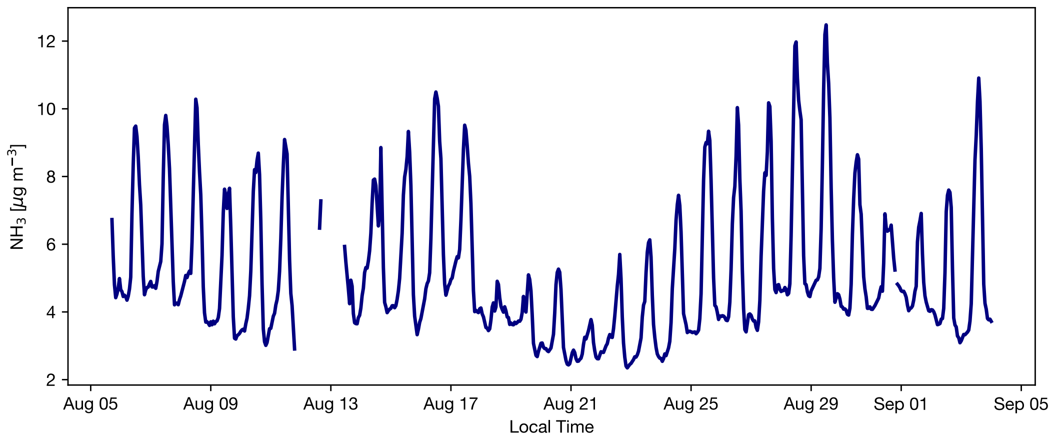

Figure S10: **Time series of NH_3 measurements during the RECAP campaign (2021).** Data were collected from a Picarro SI2103 Gas Concentration analyzer.

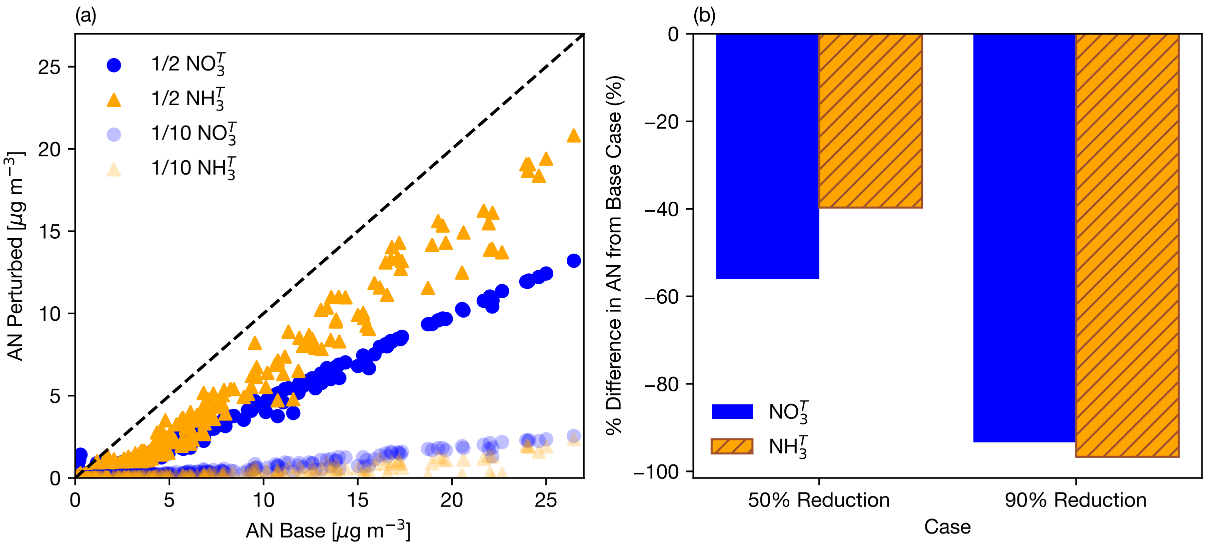

Figure S11: **Nitrate sensitivity to precursor reductions.** ISORROPPIA-II simulations are run for reduced total NO_3 and NH_3 concentrations, and the reduced total NO_3 and NH_4 cases are compared against the base case simulation. The campaign-wide results are show in (**a**) and the campaign average differences to the base simulation are shown in (**b**). Data are 1-hr time average.

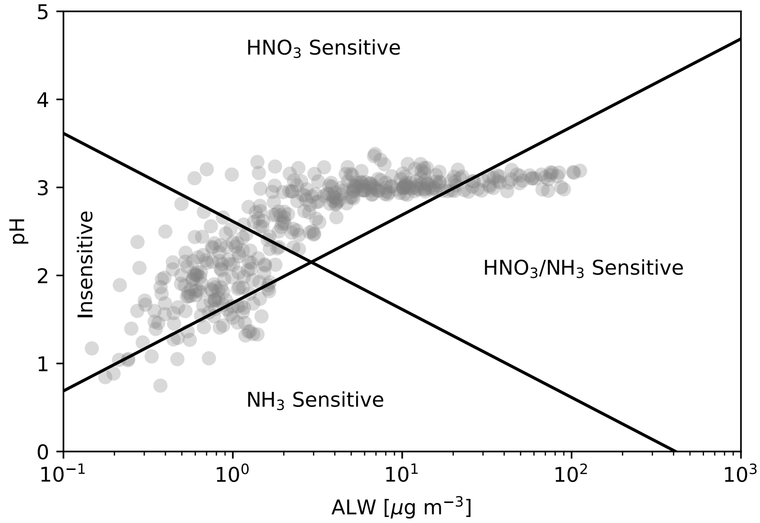

Figure S12: **Sensitivity regime for ammonium nitrate aerosol during RECAP.** Each data point is from the base case ISORROPPIA run of the RECAP campaign, and the sensitivity regime is identified after Nenes et al. (*26*). The characteristic acidity curves are calculated from the campaign average aerosol liquid water predicted by ISORROPPIA-II. Data are 1-hr time average.

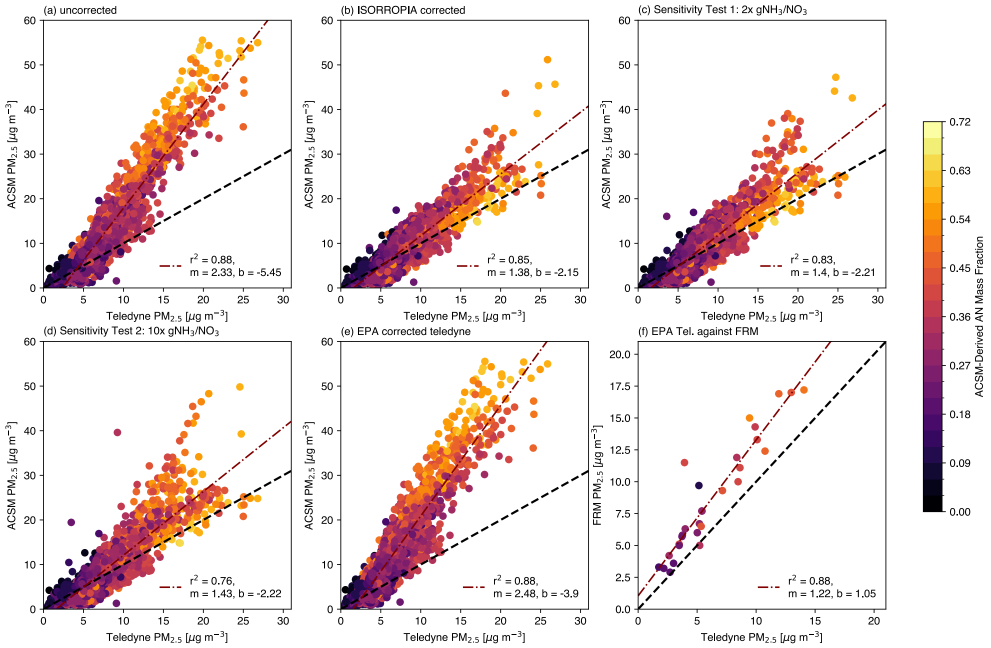

Figure S13: **Comparison of various $PM_{2.5}$ measurements in Pasadena during the Spring of 2023 to the Teledyne (FEM). (a)-(b)** are reproduced from the Main Text. **(c)-(d)** are the sensitivity studies for the ISORROPIA corrections. **(e)-(f)** adjust the Teledyne by the EPA/Teledyne correction equations released in May of 2024.

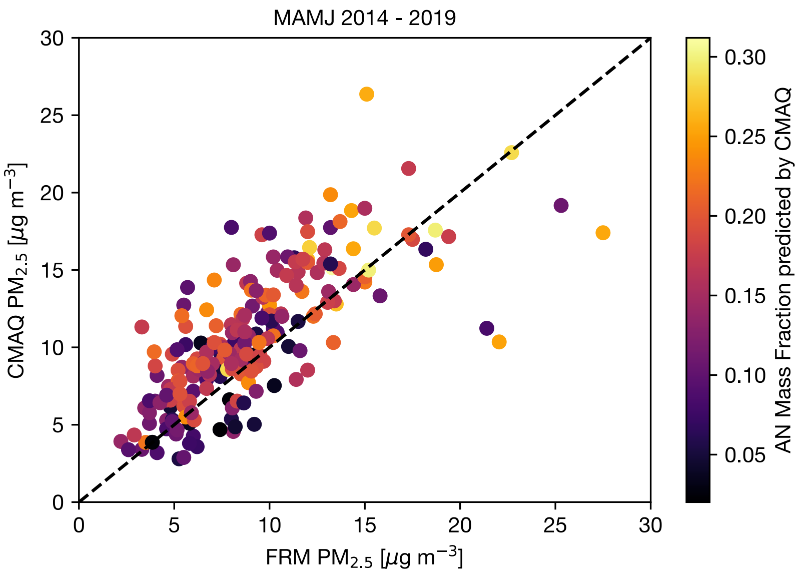

Figure S14: **Scatter plot of time series measurements of FRM $PM_{2.5}$ in Pasadena and CMAQ output for Pasadena from 2014 to 2019.** Data are for the months of March, April, May, and June and colored by the predicted mass fraction of ammonium nitrate (AN) reported by CMAQ. Each data point represents a single 24 hr period (when an FRM measurement was made, which is not every day). The dashed line represents 1:1.

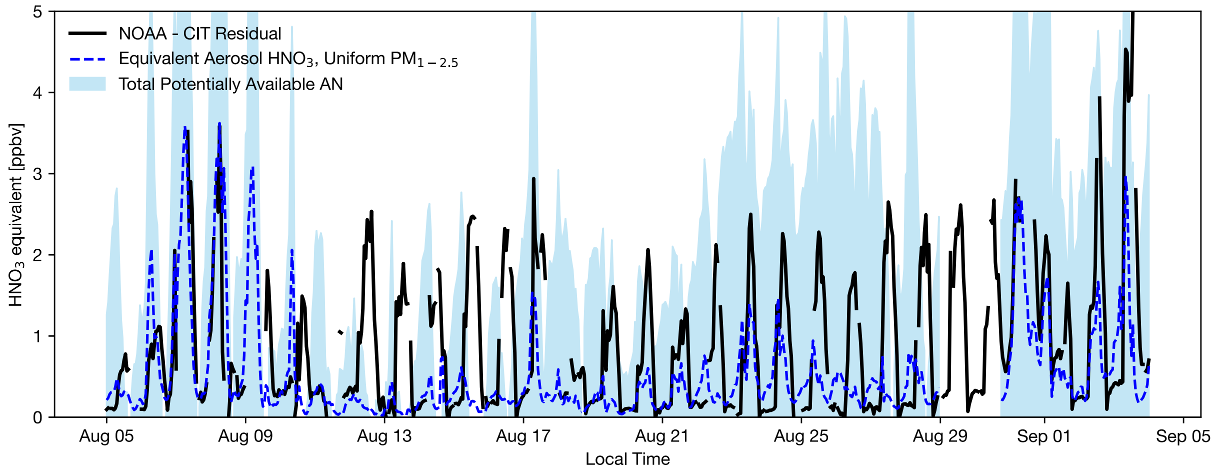

Figure S15: **HNO_3 measurements from the RECAP-CA campaign.** The solid black line represents the difference between CIMS HNO_3 measurements made by Caltech (CIT) and NOAA during the RECAP-CA campaign. The dashed blue line represents the equivalent aerosol HNO_3 laden in the $PM_{2.5}$ assuming the $PM_{1-2.5}$ is uniform to the PM_1 AMS composition. The shaded region represents this same quantity if the $PM_{1-2.5}$ was constituted completely of ammonium nitrate. Details of this calculation can be found in Section 1.3.

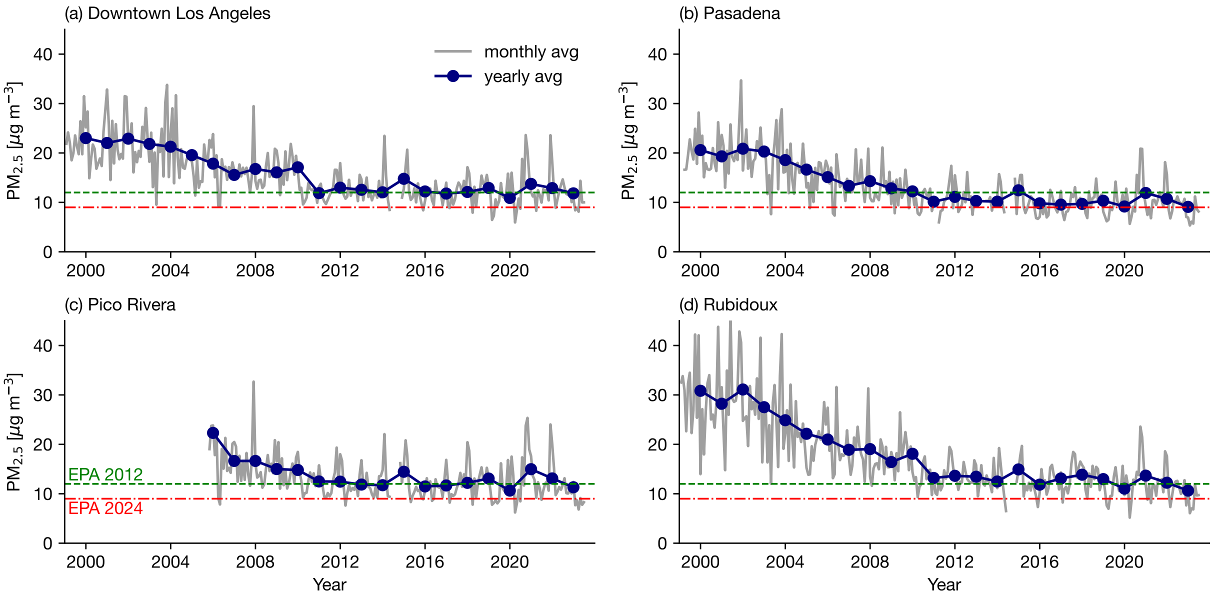

Figure S16: **Time series of $PM_{2.5}$ measured at various SCAQMD locations, aggregated to monthly (grey) and yearly (navy) averages.** The green dashed line represents the EPA's 2012 NAAQS for annual-average PM (12 μg m^{-3}) and the red dash-dot line represents the EPA's 2024 strengthening of that standard (9 μg m^{-3}).

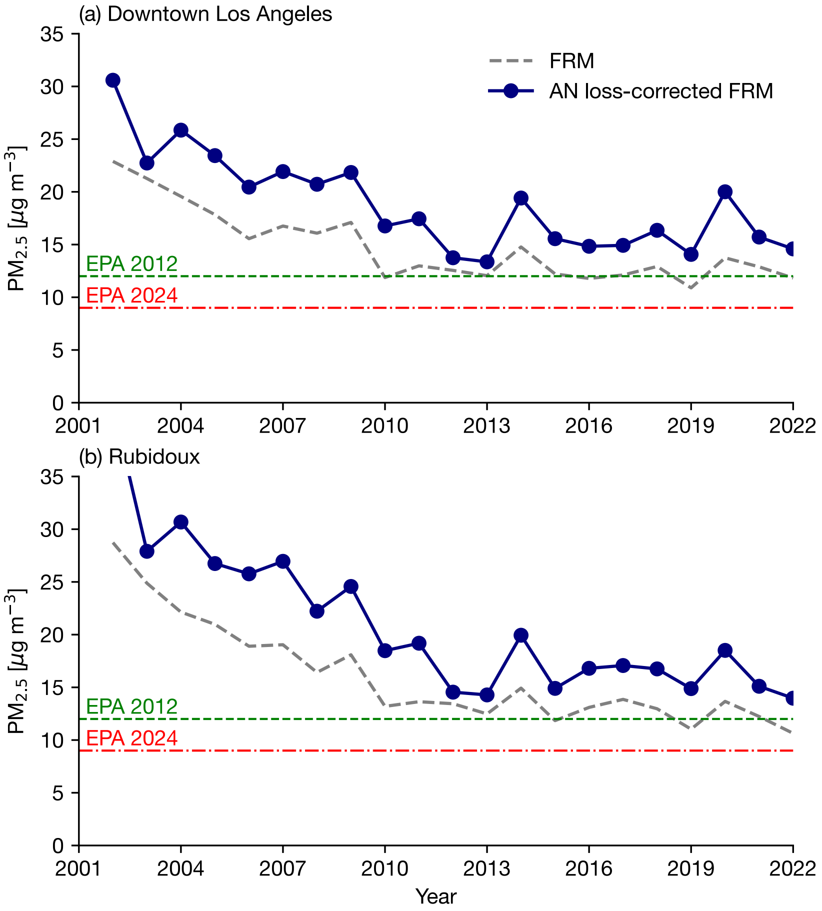

Figure S17: **Time series of $PM_{2.5}$ measured at various SCAQMD locations, corrected for the loss of ammonium nitrate.** The gray (dashed) is the uncorrected FRM yearly average while the blue (solid with marker) is the loss-corrected yearly-average. The green dashed line represents the EPA's 2012 NAAQS for annual-average PM (12 μg m^{-3}) and the red dash-dot line represents the EPA's 2024 strengthening of that standard (9 μg m^{-3}).

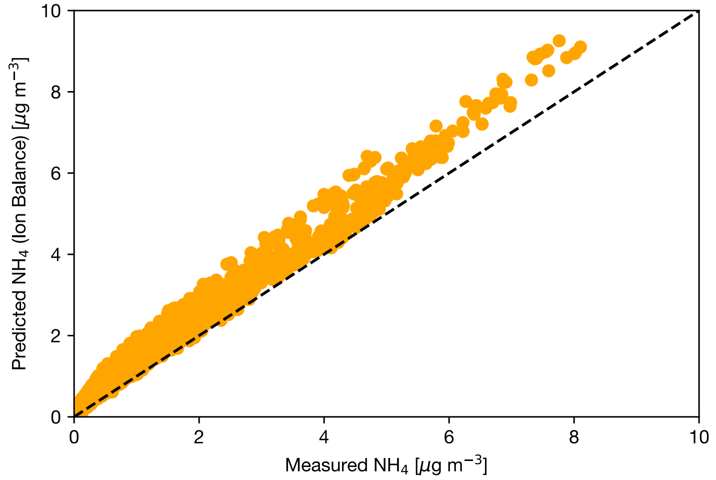

Figure S18: **Ion balance for the measurements in springtime Pasadena 2023.** Predicted NH_4^+ is calculated from the sum Cl^- + NO_3^- + $2SO_4^{2-}$. Each data point is an hourly average and the dashed line represents 1:1.

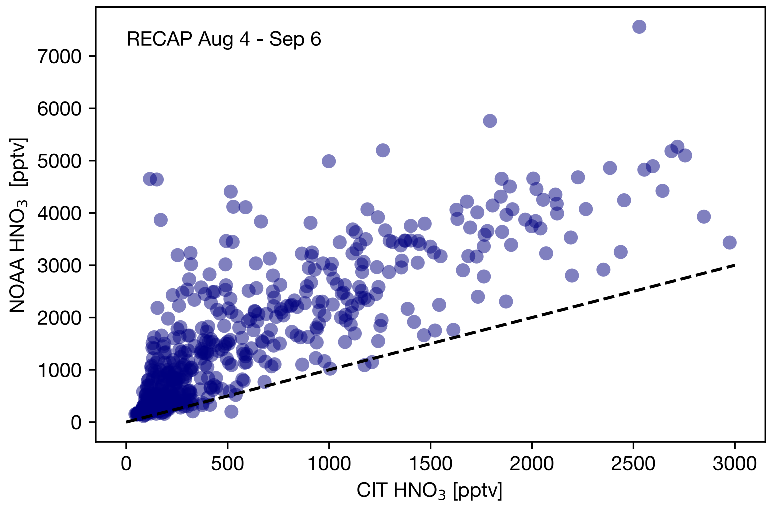

Figure S19: **HNO_3 CIMS data from the RECAP campaign.**. Scatter plot of HNO_3 measurements made by CIMS from Caltech (CIT) and NOAA, resampled to an hourly timescale. The dashed line represents 1:1.

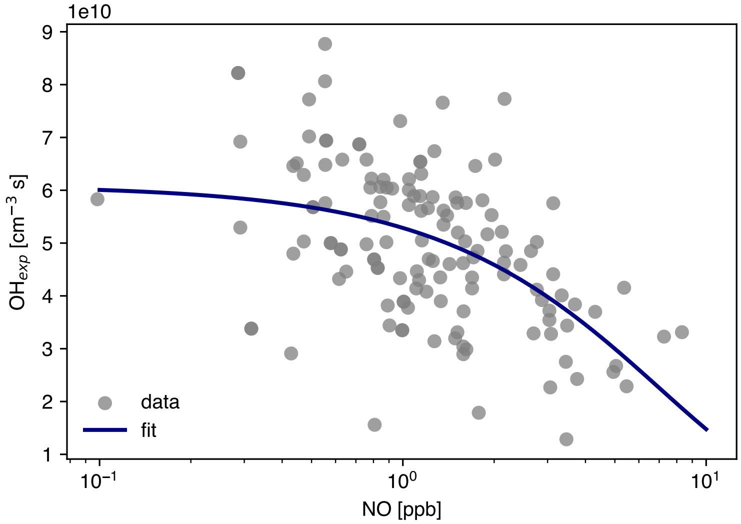

Figure S20: **Fit estimation for OH exposure.** OH exposure data are compiled at midafternoon (2-3 PM) from the CalNex (2010), LAAQC (2022), and CalNexT (2022) campaigns. NO data are from SCAQMD and represent afternoon averages.

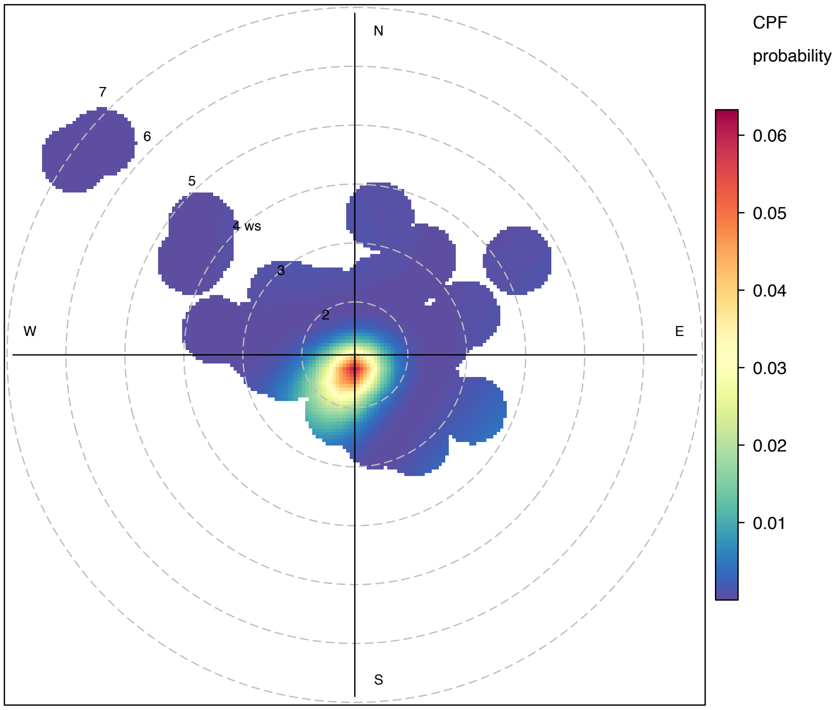

CPF at the 90th percentile (=8.5)

Figure S21: **CPF plot of the 90th percentile ($\geq$ 8.5 μg m^{-3}) for nighttime ammonium nitrate during the Pasadena 2023 campaign.** Wind speed is the radial axis, and the color corresponds to the CPF probability for ammonium nitrate.

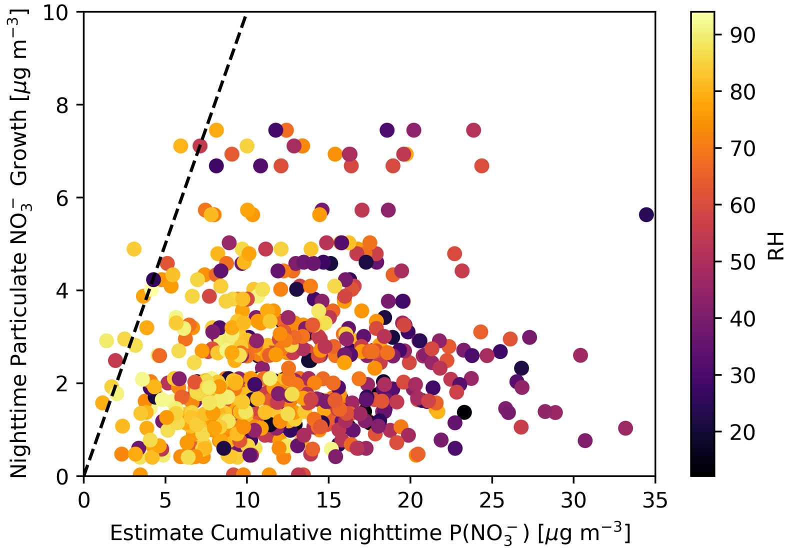

Figure S22: **Scatter plot of estimated nighttime production of nitrate aerosol and produced nitrate aerosol predicted by CMAQ.** Each data point represents a single evening in spring of the time period 2014 to 2019. Data points are colored by mean nighttime relative humidity.

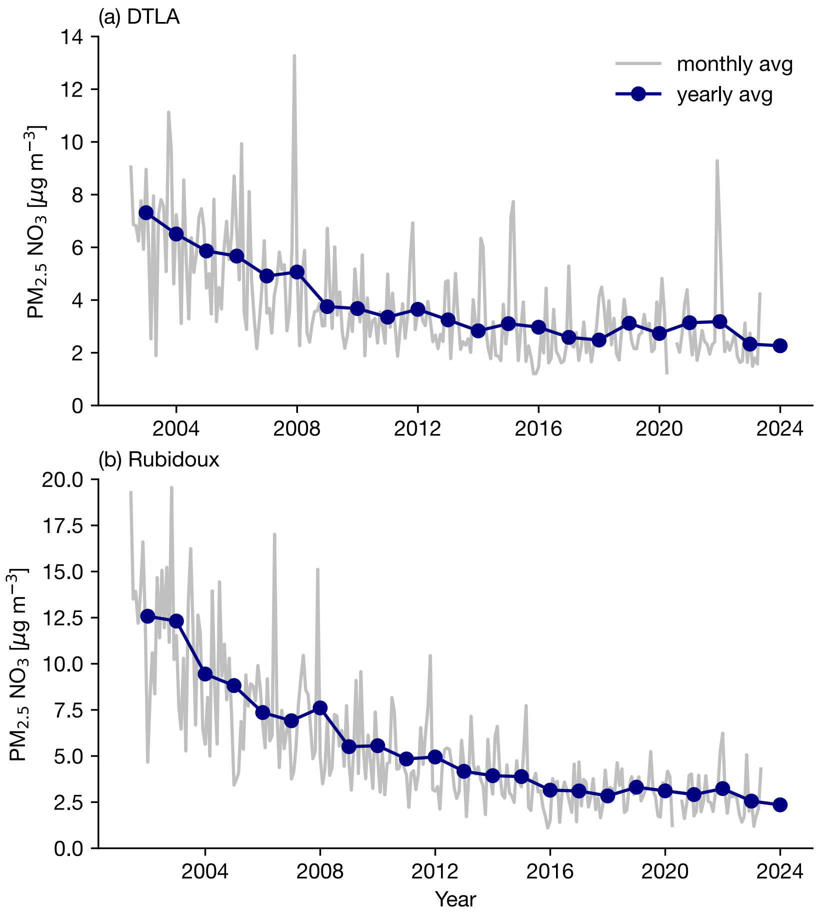

Figure S23: **Trends in $PM_{2.5}$ NO_3^- in Southern California.** Data are from two CSN (Chemical Speciation Network) sites: downtown Los Angeles (DTLA) and Riverside-Rubidoux. Data are aggregated to monthly (grey) and yearly (navy) averages.

REFERENCES AND NOTES

1. EPA, $PM_{2.5}$ (2012) Designated Area/State Information (2024), https://www3.epa.gov/airquality/greenbook/kbtc.html.

2. EPA, National Ambient Air Quality Standards (NAAQS) Table (2024), https://epa.gov/criteria-air-pollutants/naaqs-table.

3. D. D. Parrish, J. Xu, B. Croes, M. Shao, Air quality improvement in Los Angeles—Perspectives for developing cities. *Front. Environ. Sci. Eng.* **10**, 11 (2016).

4. E. A. Pennington, K. M. Seltzer, B. N. Murphy, M. Qin, J. H. Seinfeld, H. O. T. Pye, Modeling secondary organic aerosol formation from volatile chemical products. *Atmos. Chem. Phys.* **21**, 18247–18261 (2021).

5. P. L. Hayes, A. M. Ortega, M. J. Cubison, K. D. Froyd, Y. Zhao, S. S. Cliff, W. W. Hu, D. W. Toohey, J. H. Flynn, B. L. Lefer, N. Grossberg, S. Alvarez, B. Rappenglück, J. W. Taylor, J. D. Allan, J. S. Holloway, J. B. Gilman, W. C. Kuster, J. A. de Gouw, P. Massoli, X. Zhang, J. Liu, R. J. Weber, A. L. Corrigan, L. M. Russell, G. Isaacman, D. R. Worton, N. M. Kreisberg, A. H. Goldstein, R. Thalman, E. M. Waxman, R. Volkamer, Y. H. Lin, J. D. Surratt, T. E. Kleindienst, J. H. Offenberg, S. Dusanter, S. Griffith, P. S. Stevens, J. Brioude, W. M. Angevine, J. L. Jimenez, Organic aerosol composition and sources in Pasadena, California, during the 2010 CalNex campaign. *J. Geophys. Res. Atmos.* **118**, 9233–9257 (2013).

6. B. C. McDonald, J. A. de Gouw, J. B. Gilman, S. H. Jathar, A. Akherati, C. D. Cappa, J. L. Jimenez, J. Lee-Taylor, P. L. Hayes, S. A. McKeen, Y. Y. Cui, S. W. Kim, D. R. Gentner, G. Isaacman-VanWertz, A. H. Goldstein, R. A. Harley, G. J. Frost, J. M. Roberts, T. B. Ryerson, M. Trainer, Volatile chemical products emerging as largest petrochemical source of urban organic emissions. *Science* **359**, 760–764 (2018).

7. C. M. Nussbaumer, R. C. Cohen, Impact of OA on the temperature dependence of $PM_{2.5}$ in the Los Angeles Basin. *Environ. Sci. Technol.* **55**, 3549–3558 (2021).

8. S. Gu, A. Guenther, C. Faiola, Effects of anthropogenic and biogenic volatile organic compounds on Los Angeles air quality. *Environ. Sci. Technol.* **55**, 12191–12201 (2021).

9. Y. Zhao, D. S. Tkacik, A. A. May, N. M. Donahue, A. L. Robinson, Mobile sources are still an important source of secondary organic aerosol and fine particulate matter in the Los Angeles region. *Environ. Sci. Technol.* **56**, 15328–15336 (2022).

10. E. Y. Pfannerstill, C. Arata, Q. Zhu, B. C. Schulze, R. Ward, R. Woods, C. Harkins, R. H. Schwantes, J. H. Seinfeld, A. Bucholtz, R. C. Cohen, A. H. Goldstein, Temperature-dependent emissions dominate aerosol and ozone formation in Los Angeles. *Science* **384**, 1324–1329 (2024).

11. S. Hasheminassab, N. Daher, A. Saffari, D. Wang, B. D. Ostro, C. Sioutas, Spatial and temporal variability of sources of ambient fine particulate matter $PM_{2.5}$ in California. *Atmos. Chem. Phys.* **14**, 12085–12097 (2014).

12. M. J. Kleeman, G. R. Cass, A 3D Eulerian source-oriented model for an externally mixed aerosol. *Environ. Sci. Technol.* **35**, 4834–4848 (2001).

13. J. B. Nowak, J. A. Neuman, R. Bahreini, A. M. Middlebrook, J. S. Holloway, S. A. McKeen, D. D. Parrish, T. B. Ryerson, M. Trainer, Ammonia sources in the California South Coast Air Basin and their impact on ammonium nitrate formation. *Geophys. Res. Lett.* **39**, 2012GL051197 (2012).

14. J. H. Seinfeld, S. N. Pandis, *Atmospheric Chemistry and Physics: From Air Pollution to Climate Change* (Wiley & Sons) (2016).

15. C. C. Womack, E. E. McDuffie, P. M. Edwards, R. Bares, J. A. de Gouw, K. S. Docherty, W. P. Dubé, D. L. Fibiger, A. Franchin, J. B. Gilman, L. Goldberger, B. H. Lee, J. C. Lin, R. Long, A. M. Middlebrook, D. B. Millet, A. Moravek, J. G. Murphy, P. K. Quinn, T. P. Riedel, J. M. Roberts, J. A. Thornton, L. C. Valin, P. R. Veres, A. R. Whitehill, R. J. Wild, C. Warneke, B. Yuan, M. Baasandorj, S. S. Brown, An odd oxygen framework for wintertime ammonium nitrate aerosol pollution in urban areas: NO_x and VOC control as mitigation strategies. *Geophys. Res. Lett.* **46**, 4971–4979 (2019).

16. H. Li, Q. Zhang, B. Zheng, C. Chen, N. Wu, H. Guo, Y. Zhang, Y. Zheng, X. Li, K. He, Nitrate-driven urban haze pollution during summertime over the North China Plain. *Atmos. Chem. Phys.* **18**, 5293–5306 (2018).

17. S. Gani, S. Bhandari, S. Seraj, D. S. Wang, K. Patel, P. Soni, Z. Arub, G. Habib, L. Hildebrandt Ruiz, J. S. Apte, Submicron aerosol composition in the world's most polluted megacity: The Delhi Aerosol Supersite study. *Atmos. Chem. Phys.* **19**, 6843–6859 (2019).

18. Y. Wang, S. Xiao, Y. Zhang, H. Chang, R. V. Martin, A. van Donkelaar, A. Gaskins, Y. Liu, P. Liu, L. Shi, Long-term exposure to $PM_{2.5}$ major components and mortality in the southeastern United States. *Environ. Int.* **158**, 106969 (2022).

19. J. J. West, A. S. Ansari, S. N. Pandis, Marginal $PM_{2.5}$: Nonlinear aerosol mass response to sulfate reductions in the eastern United States. *J. Air Waste Manage. Assoc.* **49**, 1415–1424 (1999).

20. H. O. T. Pye, H. Liao, S. Wu, L. J. Mickley, D. J. Jacob, D. K. Henze, J. H. Seinfeld, Effect of changes in climate and emissions on future sulfate-nitrate-ammonium aerosol levels in the United States. *J. Geophys. Res. Atmos.* **114**, 2008JD010701 (2009).

21. R. J. Weber, H. Guo, A. G. Russell, A. Nenes, High aerosol acidity despite declining atmospheric sulfate concentrations over the past 15 years. *Nat. Geosci.* **9**, 282–285 (2016).

22. S. Hasheminassab, N. Daher, B. D. Ostro, C. Sioutas, Long-term source apportionment of ambient fine particulate matter ($PM_{2.5}$) in the Los Angeles Basin: A focus on emissions reduction from vehicular sources. *Environ. Pollut.* **193**, 54–64 (2014).

23. S. E. Pusede, K. C. Duffey, A. A. Shusterman, A. Saleh, J. L. Laughner, P. J. Wooldridge, Q. Zhang, C. L. Parworth, H. Kim, S. L. Capps, L. C. Valin, C. D. Cappa, A. Fried, J. Walega, J. B. Nowak, A. J. Weinheimer, R. M. Hoff, T. A. Berkoff, A. J. Beyersdorf, J. Olson, J. H. Crawford, R. C. Cohen, On the effectiveness of nitrogen oxide reductions as a control over ammonium nitrate aerosol. *Atmos. Chem. Phys.* **16**, 2575–2596 (2016).

24. H. Guo, J. Liu, K. D. Froyd, J. M. Roberts, P. R. Veres, P. L. Hayes, J. L. Jimenez, A. Nenes, R. J. Weber, Fine particle pH and gas-particle phase partitioning of inorganic species in Pasadena, California, during the 2010 CalNex campaign. *Atmos. Chem. Phys.* **17**, 5703–5719 (2017).

25. H. Guo, R. Otjes, P. Schlag, A. Kiendler-Scharr, A. Nenes, R. J. Weber, Effectiveness of ammonia reduction on control of fine particle nitrate. *Atmos. Chem. Phys.* **18**, 12241–12256 (2018).

26. A. Nenes, S. N. Pandis, R. J. Weber, A. Russell, Aerosol pH and liquid water content determine when particulate matter is sensitive to ammonia and nitrate availability. *Atmos. Chem. Phys.* **20**, 3249–3258 (2020).

27. S. Zhai, D. J. Jacob, X. Wang, Z. Liu, T. Wen, V. Shah, K. Li, J. M. Moch, K. H. Bates, S. Song, L. Shen, Y. Zhang, G. Luo, F. Yu, Y. Sun, L. Wang, M. Qi, J. Tao, K. Gui, H. Xu, Q. Zhang, T. Zhao, Y. Wang, H. C. Lee, H. Choi, H. Liao, Control of particulate nitrate air pollution in China. *Nat. Geosci.* **14**, 389–395 (2021).

28. L. D. Schiferl, C. L. Heald, J. B. Nowak, J. S. Holloway, J. A. Neuman, R. Bahreini, I. B. Pollack, T. B. Ryerson, C. Wiedinmyer, J. G. Murphy, An investigation of ammonia and inorganic particulate matter in California during the CalNex campaign. *J. Geophys. Res. Atmos.* **119**, 1883–1902 (2014).

29. J. X. Warner, R. R. Dickerson, Z. Wei, L. L. Strow, Y. Wang, Q. Liang, Increased atmospheric ammonia over the world's major agricultural areas detected from space. *Geophys. Res. Lett.* **44**, 2875–2884 (2017).

30. A. M. Burns, G. Chandler, K. J. Dunham, A. G. Carlton, Data gap: Air quality networks miss air pollution from concentrated animal feeding operations. *Environ. Sci. Technol.* **57**, 20718–20725 (2023).

31. X. Zhang, P. H. McMurry, Evaporative losses of fine particulate nitrates during sampling. *Atmos. Environ.* **26**, 3305–3312 (1992).

32. S. Hering, G. Cass, The magnitude of bias in the measurement of $PM_{2.5}$ arising from volatilization of particulate nitrate from teflon filters. *J. Air Waste Manag.. Assoc.* **49**, 725–733 (1999).

33. B. Appel, Y. Tokiwa, M. Haik, Sampling of nitrates in ambient air. *Atmos. Environ.* **15**, 283–289 (1981).

34. X. Q. Zhang, P. H. McMurry, Theoretical analysis of evaporative losses from impactor and filter deposits. *Atmos. Environ.* **21**, 1779–1789 (1987).

35. Y. T. T. Chiu, A. G. Carlton, Aerosol thermodynamics: Nitrate loss from regulatory $PM_{2.5}$ filters in California. *ACS EST Air* **1**, 25–32 (2023).

36. S. S. Brown, H. Stark, T. B. Ryerson, E. J. Williams, D. K. Nicks Jr., M. Trainer, F. C. Fehsenfeld, A. R. Ravishankara, Nitrogen oxides in the nocturnal boundary layer: Simultaneous in situ measurements of NO_3, N_2O_5, NO_2, NO, and O_3. *J. Geophys. Res. Atmos.* **108**, 2002JD002917 (2003).

37. P. Van Rooy, A. Tasnia, B. Barletta, R. Buenconsejo, J. D. Crounse, C. M. Kenseth, S. Meinardi, S. Murphy, H. Parker, B. Schulze, J. H. Seinfeld, P. O. Wennberg, D. R. Blake, K. C. Barsanti, Observations of volatile organic compounds in the Los Angeles Basin during COVID-19. *ACS Earth Space Chem.* **5**, 3045–3055 (2021).

38. A. R. Jensen, M. A. Morris, B. C. Schulze, A. C. Bradley, L. D. Anderson, O. J. Jenks, W. D. Dresser, K. Ball, R. X. Ward, D. A. Day, J. D. Crounse, S. Meinardi, B. Barletta, D. R. Blake, J. H. Seinfeld, P. O. Wennberg, J. L. Jimenez, J. A. de Gouw, Emissions and chemistry of volatile organic compounds in the Los Angeles Basin in summer 2022. *J. Geophys. Res. Atmos.* **129**, e2024JD041812 (2024).

39. I. B. Pollack, T. B. Ryerson, M. Trainer, J. A. Neuman, J. M. Roberts, D. D. Parrish, Trends in ozone, its precursors, and related secondary oxidation products in Los Angeles, California: A synthesis of measurements from 1960 to 2010. *J. Geophys. Res. Atmos.* **118**, 5893–5911 (2013).

40. N. L. Ng, S. C. Herndon, A. Trimborn, M. R. Canagaratna, P. L. Croteau, T. B. Onasch, D. Sueper, D. R. Worsnop, Q. Zhang, Y. L. Sun, J. T. Jayne, An aerosol chemical speciation monitor (ACSM) for routine monitoring of the composition and mass concentrations of ambient aerosol. *Aerosol Sci. Tech.* **45**, 780–794 (2011).

41. R. Fröhlich, M. J. Cubison, J. G. Slowik, N. Bukowiecki, A. S. H. Prévôt, U. Baltensperger, J. Schneider, J. R. Kimmel, M. Gonin, U. Rohner, D. R. Worsnop, J. T. Jayne, The ToF-ACSM: A portable aerosol chemical speciation monitor with TOFMS detection. *Atmos. Meas. Tech.* **6**, 3225–3241 (2013).

42. W. Xu, P. Croteau, L. Williams, M. Canagaratna, T. Onasch, E. Cross, X. Zhang, W. Robinson, D. Worsnop, J. Jayne, Laboratory characterization of an aerosol chemical speciation monitor with $PM_{2.5}$ measurement capability. *Aerosol Sci. Tech.* **51**, 69–83 (2017).

43. A. G. Russell, G. J. McRae, G. R. Cass, Mathematical modeling of the formation and transport of ammonium nitrate aerosol. *Atmos. Environ.* **17**, 949–964 (1983).

44. S. P. Hersey, J. S. Craven, K. A. Schilling, A. R. Metcalf, A. Sorooshian, M. N. Chan, R. C. Flagan, J. H. Seinfeld, The Pasadena Aerosol Characterization Observatory (PACO): Chemical and physical analysis of the Western Los Angeles basin aerosol. *Atmos. Chem. Phys.* **11**, 7417–7443 (2011).

45. S. P. Hersey, J. S. Craven, A. R. Metcalf, J. Lin, T. Lathem, K. J. Suski, J. F. Cahill, H. T. Duong, A. Sorooshian, H. H. Jonsson, M. Shiraiwa, A. Zuend, A. Nenes, K. A. Prather, R. C. Flagan, J. H. Seinfeld, Composition and hygroscopicity of the Los Angeles aerosol: CalNex. *J. Geophys. Res. Atmos.* **118**, 3016–3036 (2013).

46. P. Sun, R. N. Farley, L. Li, D. Srivastava, C. R. Niedek, J. Li, N. Wang, C. D. Cappa, S. E. Pusede, Z. Yu, P. Croteau, Q. Zhang, $PM_{2.5}$ composition and sources in the San Joaquin Valley of California: A long-term study using ToF-ACSM with the capture vaporizer. *Environ. Pollut.* **292**, 118254 (2022).

47. T. Joo, Y. Chen, W. Xu, P. Croteau, M. R. Canagaratna, D. Gao, H. Guo, G. Saavedra, S. S. Kim, Y. Sun, R. Weber, J. Jayne, N. L. Ng, Evaluation of a new aerosol chemical speciation monitor (ACSM) system at an urban site in Atlanta, GA: The use of capture vaporizer and $PM_{2.5}$ inlet. *ACS Earth Space Chem.* **5**, 2565–2576 (2021).

48. M. E. Bassett, J. H. Seinfeld, Atmospheric equilibrium model of sulfate and nitrate aerosols–II. Particle size analysis. *Atmos. Environ.* **18**, 1163–1170 (1984).

49. D.-Y. Liu, K. A. Prather, S. V. Hering, Variations in the size and chemical composition of nitrate-containing particles in Riverside, CA. *Aerosol Sci. Technol.* **33**, 71–86 (2000).

50. L. S. Hughes, J. O. Allen, L. G. Salmon, P. R. Mayo, R. J. Johnson, G. R. Cass, Evolution of nitrogen species air pollutants along trajectories crossing the Los Angeles area. *Environ. Sci. Technol.* **36**, 3928–3935 (2002).

51. Mariam, M. Joshi, P. Khandare, A. Koli, A. Khan, B. K. Sapra, Influence of sheath air humidity on measurement of particle size distribution by scanning mobility particle sizer. *J. Aerosol Sci.* **111**, 18–25 (2017).

52. H. Wang, K. Lu, X. Chen, Q. Zhu, Q. Chen, S. Guo, M. Jiang, X. Li, D. Shang, Z. Tan, Y. Wu, Z. Wu, Q. Zou, Y. Zheng, L. Zeng, T. Zhu, M. Hu, Y. Zhang, High N_2O_5 concentrations observed in urban Beijing: Implications of a large nitrate formation pathway. *Environ. Sci. Technol. Lett.* **4**, 416–420 (2017).

53. EPA, Update of $PM_{2.5}$ Data From T640/T640X PM Mass Monitors (2024).

54. Teledyne-API, Development of an FRM alignment factor for the Teledyne API (TAPI) Model T640/x Instruments (2024).

55. R. W. Long, S. P. Urbanski, E. Lincoln, M. Colón, S. Kaushik, J. D. Krug, R. W. Vanderpool, M. S. Landis, Summary of $PM_{2.5}$ measurement artifacts associated with the Teledyne T640 PM mass monitor under controlled chamber experimental conditions using polydisperse ammonium sulfate aerosols and biomass smoke. *J. Air Waste Manag. Assoc.* **73**, 295–312 (2023).

56. A. Van Donkelaar, M. S. Hammer, L. Bindle, M. Brauer, J. R. Brook, M. J. Garay, N. C. Hsu, O. V. Kalashnikova, R. A. Kahn, C. Lee, R. C. Levy, A. Lyapustin, A. M. Sayer, R. V. Martin, Monthly global estimates of fine particulate matter and their uncertainty. *Environ. Sci. Technol.* **55**, 15287–15300 (2021).

57. EPA, EQUATES: EPA's Air QUAlity TimE Series Project (2021).

58. K. K. Barkjohn, B. Gantt, A. L. Clements, Development and application of a United States-wide correction for $PM_{2.5}$ data collected with the PurpleAir sensor. *Atmos. Meas. Tech.* **14**, 4617–4637 (2021).

59. A. Datta, A. Saha, M. L. Zamora, C. Buehler, L. Hao, F. Xiong, D. R. Gentner, K. Koehler, Statistical field calibration of a low-cost $PM_{2.5}$ monitoring network in Baltimore. *Atmos. Environ.* **242**, 117761 (2020).

60. M. Levy Zamora, F. Xiong, D. Gentner, B. Kerkez, J. Kohrman-Glaser, K. Koehler, Field and laboratory evaluations of the low-cost Plantower particulate matter sensor. *Environ. Sci. Technol.* **53**, 838–849 (2019).

61. W. Nie, T. Wang, X. Gao, R. K. Pathak, X. Wang, R. Gao, Q. Zhang, L. Yang, W. Wang, Comparison among filter-based, impactor-based and continuous techniques for measuring atmospheric fine sulfate and nitrate. *Atmos. Environ.* **44**, 4396–4403 (2010).

62. B. A. Nault, P. Campuzano-Jost, D. A. Day, H. Guo, D. S. Jo, A. V. Handschy, D. Pagonis, J. C. Schroder, M. K. Schueneman, M. J. Cubison, J. E. Dibb, A. Hodzic, W. Hu, B. B. Palm, J. L. Jimenez, Interferences with aerosol acidity quantification due to gas-phase ammonia uptake onto acidic sulfate filter samples. *Atmos. Meas. Tech.* **13**, 6193–6213 (2020).

63. B. Appel, S. Wall, Y. Tokiwa, M. Haik, Simultaneous nitric acid, particulate nitrate and acidity measurements in ambient air. *Atmos. Environ.* **14**, 549–554 (1980).

64. SCAQMD, 2022 Air Quality Management Plan (2022).

65. R. Burnett, H. Chen, M. Szyszkowicz, N. Fann, B. Hubbell, C. A. Pope III, J. S. Apte, M. Brauer, A. Cohen, S. Weichenthal, J. Coggins, Q. di, B. Brunekreef, J. Frostad, S. S. Lim, H.

Kan, K. D. Walker, G. D. Thurston, R. B. Hayes, C. C. Lim, M. C. Turner, M. Jerrett, D. Krewski, S. M. Gapstur, W. R. Diver, B. Ostro, D. Goldberg, D. L. Crouse, R. V. Martin, P. Peters, L. Pinault, M. Tjepkema, A. van Donkelaar, P. J. Villeneuve, A. B. Miller, P. Yin, M. Zhou, L. Wang, N. A. H. Janssen, M. Marra, R. W. Atkinson, H. Tsang, T. Quoc Thach, J. B. Cannon, R. T. Allen, J. E. Hart, F. Laden, G. Cesaroni, F. Forastiere, G. Weinmayr, A. Jaensch, G. Nagel, H. Concin, J. V. Spadaro, Global estimates of mortality associated with long-term exposure to outdoor fine particulate matter. *Proc. Natl. Acad. Sci. U.S.A.* **115**, 9592–9597 (2018).

66. M. M. H. El-Sayed, D. Amenumey, C. J. Hennigan, Drying-induced evaporation of secondary organic aerosol during summer. *Environ. Sci. Technol.* **50**, 3626–3633 (2016).

67. Q. Di, H. Amini, L. Shi, I. Kloog, R. Silvern, J. Kelly, M. B. Sabath, C. Choirat, P. Koutrakis, A. Lyapustin, Y. Wang, L. J. Mickley, J. Schwartz, An ensemble-based model of $PM_{2.5}$ concentration across the contiguous United States with high spatiotemporal resolution. *Environ. Int.* **130**, 104909 (2019).

68. T. S. Carter, G. H. Kerr, H. Amini, R. V. Martin, U. Ovienmhada, J. Schwartz, A. van Donkelaar, S. Anenberg, $PM_{2.5}$ data inputs alter identification of disadvantaged communities. *Environ. Res. Lett.* **18**, 114008 (2023).

69. G. H. Kerr, A. van Donkelaar, R. V. Martin, M. Brauer, K. Bukart, S. Wozniak, D. L. Goldberg, S. C. Anenberg, Increasing racial and ethnic disparities in ambient air pollution-attributable morbidity and mortality in the United States. *Environ. Health Perspect.* **132**, 037002 (2024).

70. A. M. Middlebrook, R. Bahreini, J. L. Jimenez, M. R. Canagaratna, Evaluation of composition-dependent collection efficiencies for the aerodyne aerosol mass spectrometer using field data. *Aerosol Sci. Tech.* **46**, 258–271 (2012).

71. W. Hu, P. Campuzano-Jost, D. A. Day, B. A. Nault, T. Park, T. Lee, A. Pajunoja, A. Virtanen, P. Croteau, M. R. Canagaratna, J. T. Jayne, D. R. Worsnop, J. L. Jimenez, Ambient quantification and size distributions for organic aerosol in aerosol mass spectrometers with the new capture vaporizer. *ACS Earth Space Chem.* **4**, 676–689 (2020).

72. M. R. Canagaratna, J. L. Jimenez, J. H. Kroll, Q. Chen, S. H. Kessler, P. Massoli, L. Hildebrandt Ruiz, E. Fortner, L. R. Williams, K. R. Wilson, J. D. Surratt, N. M. Donahue, J. T. Jayne, D. R. Worsnop, Elemental ratio measurements of organic compounds using aerosol mass spectrometry: Characterization, improved calibration, and implications. *Atmos. Chem. Phys.* **15**, 253–272 (2015).

73. M. Kuwata, S. R. Zorn, S. T. Martin, Using elemental ratios to predict the density of organic material composed of carbon, hydrogen, and oxygen. *Environ. Sci. Technol.* **46**, 787–794 (2012).

74. K. Ardon-Dryer, Y. Dryer, J. N. Williams, N. Moghimi, Measurements of $PM_{2.5}$ with PurpleAir under atmospheric conditions. *Atmos. Meas. Tech.* **13**, 5441–5458 (2020).

75. J. D. Crounse, K. A. McKinney, A. J. Kwan, P. O. Wennberg, Measurement of gas-phase hydroperoxides by chemical ionization mass spectrometry. *Anal. Chem.* **78**, 6726–6732 (2006).

76. M. A. Robinson, J. A. Neuman, L. G. Huey, J. M. Roberts, S. S. Brown, P. R. Veres, Temperature-dependent sensitivity of iodide chemical ionization mass spectrometers. *Atmos. Meas. Tech.* **15**, 4295–4305 (2022).

77. F. C. Fehsenfeld, L. G. Huey, D. T. Sueper, R. B. Norton, E. J. Williams, F. L. Eisele, R. L. Mauldin III, D. J. Tanner, Ground-based intercomparison of nitric acid measurement techniques. *J. Geophys. Res. Atmos.* **103**, 3343–3353 (1998).

78. J. A. De Gouw, J. A. de Gouw, J. B. Gilman, S.-W. Kim, S. L. Alvarez, S. Dusanter, M. Graus, S. M. Griffith, G. I.-V. Wertz, W. C. Kuster, B. L. Lefer, B. M. Lerner, B. C. M. Donald, B. Rappenglück, J. M. Roberts, P. S. Stevens, J. Stutz, R. Thalman, P. R. Veres, R. Volkamer, C. Warneke, R. A. Washenfelder, C. J. Young, Chemistry of volatile organic compounds in the Los Angeles Basin: Formation of oxygenated compounds and determination of emission ratios. *J. Geophys. Res. Atmos.* **122**, 2298–2319 (2017).

79. C. Fountoukis, A. Nenes, ISORROPIA II: A computationally efficient thermodynamic equilibrium model for K^+-Ca^{2+}-Mg^{2+}-NH_4^+-Na^+-SO_4^{2-}-NO_3^--Cl^--H_2O aerosols. *Atmos. Chem. Phys.* **7**, 4639–4659 (2007).

80. S. Song, M. Gao, W. Xu, J. Shao, G. Shi, S. Wang, Y. Wang, Y. Sun, M. B. McElroy, Fine-particle pH for Beijing winter haze as inferred from different thermodynamic equilibrium models. *Atmos. Chem. Phys.* **18**, 7423–7438 (2018).

81. A. S. Wexler, J. H. Seinfeld, Analysis of aerosol ammonium nitrate: Departures from equilibrium during SCAQS. *Atmos. Environ.* **26**, 579–591 (1992).

82. P. Acharja, S. D. Ghude, B. Sinha, M. Barth, G. Govardhan, R. Kulkarni, V. Sinha, R. Kumar, K. Ali, I. Gultepe, J. E. Petit, M. N. Rajeevan, Thermodynamical framework for effective mitigation of high aerosol loading in the Indo-Gangetic Plain during winter. *Sci. Rep.* **13**, 13667 (2023).

83. Y. Wang, Q. Q. Zhang, K. He, Q. Zhang, L. Chai, Sulfate-nitrate-ammonium aerosols over China: Response to 2000-2015 emission changes of sulfur dioxide, nitrogen oxides, and ammonia. *Atmos. Chem. Phys.* **13**, 2635–2652 (2013).

84. Q. Zhu, J. L. Laughner, R. C. Cohen, Estimate of OH trends over one decade in North American cities. *Proc. Natl. Acad. Sci. U.S.A.* **119**, e2117399119 (2022).

85. HEI panel on the health effects of long-term exposure to traffic-related air pollution, Systematic review and meta-analysis of selected health effects of long-term exposure to traffic-related air pollution. *Special Report 23* (2022).

86. R. Wyzga, A. Rohr, Long-term particulate matter exposure: Attributing health effects to individual PM components. *J. Air Waste Manag. Assoc.* **65**, 523–543 (2015).

87. G. D. Thurston, R. T. Burnett, M. C. Turner, Y. Shi, D. Krewski, R. Lall, K. Ito, M. Jerrett, S. M. Gapstur, W. R. Diver, C. A. Pope III, Ischemic heart disease mortality and long-term exposure to source-related components of U.S. fine particle air pollution. *Environ. Health Perspect.* **124**, 785–794 (2016).

88. K. J. Colonna, P. Koutrakis, P. L. Kinney, R. M. Cooke, J. S. Evans, Mortality attributable to long-term exposure to ambient fine particulate matter: Insights from the epidemiologic evidence for understudied locations. *Environ. Sci. Technol.* **56**, 6799–6812 (2022).

89. I. Uria-Tellaetxe, D. C. Carslaw, Conditional bivariate probability function for source identification. *Environ. Model. Software* **59**, 1–9 (2014).

90. CARB, California Emissions Projection Analysis Model (CEPAM) (2019).

91. C. L. Heald, J. L. Collett Jr., T. Lee, K. B. Benedict, F. M. Schwandner, Y. Li, L. Clarisse, D. R. Hurtmans, M. van Damme, C. Clerbaux, P. F. Coheur, S. Philip, R. V. Martin, H. O. T. Pye, Atmospheric ammonia and particulate inorganic nitrogen over the United States. *Atmos. Chem. Phys.* **12**, 10295–10312 (2012).
